# Resilience of Microbial Communities after Hydrogen Peroxide Treatment of a Eutrophic Lake to Suppress Harmful Cyanobacterial Blooms

**DOI:** 10.3390/microorganisms9071495

**Published:** 2021-07-13

**Authors:** Tim Piel, Giovanni Sandrini, Gerard Muyzer, Corina P. D. Brussaard, Pieter C. Slot, Maria J. van Herk, Jef Huisman, Petra M. Visser

**Affiliations:** 1 Department of Freshwater and Marine Ecology, Institute for Biodiversity and Ecosystem Dynamics, University of Amsterdam, 1090 GE Amsterdam, The Netherlands; t.f.piel@uva.nl (T.P.); giovanni.sandrini@gmail.com (G.S.); g.muijzer@uva.nl (G.M.); corina.brussaard@nioz.nl (C.P.D.B.); p.c.slot@uva.nl (P.C.S.); m.j.vanherk@uva.nl (M.J.v.H.); j.huisman@uva.nl (J.H.); 2 Department of Marine Microbiology and Biogeochemistry, NIOZ Royal Netherland Institute for Sea Research, 1790 AB Den Burg, The Netherlands

**Keywords:** cyanobacterial blooms, 16S rRNA gene amplicon sequencing, hydrogen peroxide, microbial community, microbial diversity, resilience, ecosystem functioning, oxidative stress, lake treatment

## Abstract

Applying low concentrations of hydrogen peroxide (H_2_O_2_) to lakes is an emerging method to mitigate harmful cyanobacterial blooms. While cyanobacteria are very sensitive to H_2_O_2_, little is known about the impacts of these H_2_O_2_ treatments on other members of the microbial community. In this study, we investigated changes in microbial community composition during two lake treatments with low H_2_O_2_ concentrations (target: 2.5 mg L^−1^) and in two series of controlled lake incubations. The results show that the H_2_O_2_ treatments effectively suppressed the dominant cyanobacteria *Aphanizomenon klebahnii*, *Dolichospermum* sp. and, to a lesser extent, *Planktothrix agardhii*. Microbial community analysis revealed that several Proteobacteria (e.g., Alteromonadales, Pseudomonadales, Rhodobacterales) profited from the treatments, whereas some bacterial taxa declined (e.g., Verrucomicrobia). In particular, the taxa known to be resistant to oxidative stress (e.g., *Rheinheimera*) strongly increased in relative abundance during the first 24 h after H_2_O_2_ addition, but subsequently declined again. Alpha and beta diversity showed a temporary decline but recovered within a few days, demonstrating resilience of the microbial community. The predicted functionality of the microbial community revealed a temporary increase of anti-ROS defenses and glycoside hydrolases but otherwise remained stable throughout the treatments. We conclude that the use of low concentrations of H_2_O_2_ to suppress cyanobacterial blooms provides a short-term pulse disturbance but is not detrimental to lake microbial communities and their ecosystem functioning.

## 1. Introduction

Harmful cyanobacterial blooms often occur in eutrophic freshwater ecosystems, causing degradation of water quality and possibly ecological and economic disruption [1,2,3,4]. Many bloom-forming cyanobacteria can produce toxins which harm animals such as fish, birds and mammals, including humans, either directly after ingestion of contaminated water or indirectly via the food chain [5,6,7,8]. The widespread increase of harmful cyanobacterial blooms around the globe has been linked to eutrophication and climate change [9,10,11,12].

The most effective and most preferred long-term method to reduce cyanobacterial blooms is reduction of nutrient inputs into the water body [13,14]. Due to the difficulties in managing catchment nutrient loads [15] and internal nutrient loading from the sediment of lakes [16], it often takes a long time before nutrient reduction measures result in a decrease of cyanobacterial abundances. Hence, there is an increased interest in short-term methods to rapidly suppress cyanobacterial blooms that can complement long-term nutrient reduction strategies [4,17,18].

Addition of hydrogen peroxide (H_2_O_2_) to lakes is a promising method for rapid mitigation of cyanobacterial blooms because cyanobacteria are more sensitive to low concentrations of H_2_O_2_ than most eukaryotic organisms [19,20,21,22,23,24,25,26]. Furthermore, H_2_O_2_ is a naturally occurring compound that degrades into water and oxygen, and hence, unlike other algicides, the added H_2_O_2_ disappears within a few days [27]. The high sensitivity of cyanobacteria to H_2_O_2_ in comparison to eukaryotic phytoplankton can likely be attributed to differences in the Mehler reaction. In eukaryotic organisms, photosynthesis can lead to the production of reactive oxygen species (ROS) such as O_2_^−^ and H_2_O_2_, which are subsequently converted to water by peroxidases and catalases to protect cells against oxidative stress [28,29]. In cyanobacteria, the transfer of excess electrons generated by photosynthesis is mediated by flavodiiron proteins which produce water without the formation of O_2_^−^ or H_2_O_2_ [30,31]. Accordingly, cyanobacteria produce less H_2_O_2_ during photosynthesis, and hence they tend to be much less protected against H_2_O_2_ than eukaryotic phytoplankton [25,27].

Effects of H_2_O_2_ on cyanobacteria have been investigated mainly in small-scale laboratory experiments [32,33,34,35,36,37,38,39,40], mesocosms and pond experiments [21,22,23,24,26,41,42]. Only a few studies have investigated H_2_O_2_ treatments of entire lakes, reporting the impact on cyanobacteria, eukaryotic phytoplankton, zooplankton and macroinvertebrates at the ecosystem scale [25,43,44,45]. Generally, these studies show that cyanobacteria are indeed much more sensitive to H_2_O_2_ than most of the eukaryotic organisms.

However, what is the impact of H_2_O_2_ addition on the prokaryotic microbial communities in lakes? Several papers report on laboratory experiments investigating effects of H_2_O_2_ on specific groups of microorganisms, e.g., bacterial pathogens [46,47,48], root nodule bacteria [49,50] and different members of the human microbiome [51,52]. The effect of H_2_O_2_ on the activity and composition of natural microbial communities has been investigated in small-scale incubations with eutrophic lake water or soils [26,42,53,54]. Santos et al. [42] recently described mesocosm experiments in which H_2_O_2_ addition led to increased abundances of Firmicutes and Proteobacteria and decreased abundances of Actinobacteria, Verrucomicrobia, Planctomycetes and Chloroflexi. Yet, the impact of large-scale H_2_O_2_ treatments on the entire microbial community of lakes has, to our knowledge, not been investigated thus far. Bacteria perform important ecological functions in freshwater environments, such as the decomposition of dead organic matter and the recycling of nutrients like carbon, nitrogen and sulfur [55,56]. From a lake management perspective, it is therefore of key interest to gain a better understanding of the potential impact of H_2_O_2_ treatments on the microbial communities of lakes.

**Figure 1:**
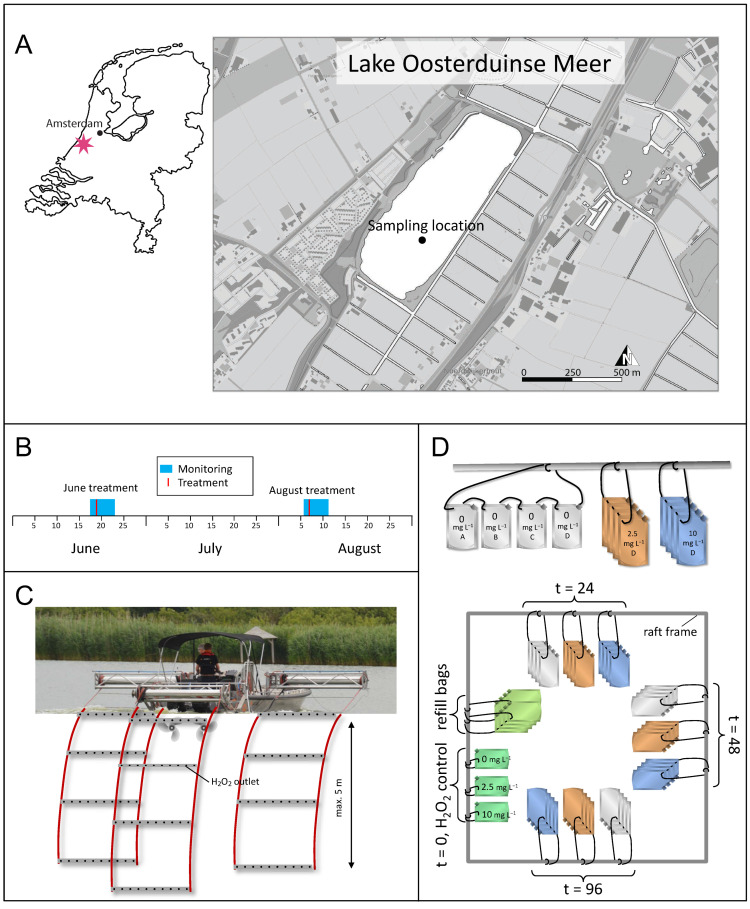
Location of the lake and experimental design. (**A**) The location of lake Oosterduinse Meer in The Netherlands and the sampling location in the lake. In June and August (**B**), H_2_O_2_ was carefully added to the top 5 m of the lake with the help of a specialized boat (**C**). Both lake treatments were accompanied by incubation experiments (**D**). The incubation bags were attached to a raft which was anchored at the sampling location in the lake. All the incubation bags were filled with surface lake water at t = −24 h and attached to the raft in groups of four replicate incubation bags per treatment condition (control, 2.5 and 10 mg L^−1^ H_2_O_2_) and sampling time point (t = 24 h, t = 48 h and t = 96 h). At the same time as the lake was treated with H_2_O_2_, the incubation bags of all the treatment conditions and sampling time points received 0 mg L^−1^ H_2_O_2_ (Milli-Q) in the control incubations (gray) and 2.5 mg L^−1^ H_2_O_2_ (orange) and 10 mg L^−1^ H_2_O_2_ (blue) in the other experimental treatments. The three incubation bags at t = 0 h (dark green) were treated with either 0, 2.5 or 10 mg L^−1^ H_2_O_2_ and immediately brought to shore and sampled 10 min after the treatment for H_2_O_2_ concentration determination as well as phytoplankton analysis. At each consecutive time point, the corresponding incubation bags of the three treatments (gray, orange and blue) were taken out of the water and brought to shore for analysis. Each mesocosm bag served as a biological replicate and was sampled only once.

This study sought to fill this gap by monitoring changes in microbial diversity and community composition using 16S rRNA gene amplicon sequencing during two H_2_O_2_ treatments of an entire lake, one in June and the other in August (Figure 1A,B). Lake treatments represent “real-life” scenarios in which microbial communities are not only affected by the treatment, but may also respond to many other factors. Without adequate controls, it may be difficult to discern to what extent the observed changes in the microbial community composition are caused by the H_2_O_2_ treatment, by natural variability of the community or by concomitant changes of other environmental drivers (e.g., changing weather conditions). Therefore, both lake treatments were accompanied by replicated in situ lake incubations with two different H_2_O_2_ concentrations and a control (without H_2_O_2_ addition). The results of our study give insight in the resistance and resilience of the microbial community after lake treatments with H_2_O_2_, the taxa that suffer or benefit from the treatment and the extent to which H_2_O_2_ impacts the functioning of microbial communities.

## 2. Materials and Methods

### 2.1. Lake, H_2_O_2_ Treatment and Sampling Information

Lake Oosterduinse Meer is located in a rural area close to the Dutch coast (52°16′55″ N, 4°30′28″ E) (Figure 1A). It covers 30 ha, has an average depth of 7 m (maximum depth of 13 m) and is stratified during summer months. The lake is connected with the surrounding flower fields with a canal system. Consequently, the lake water is hypertrophic and especially rich in phosphorus, making it an excellent environment for cyanobacterial blooms.

**Figure 2:**
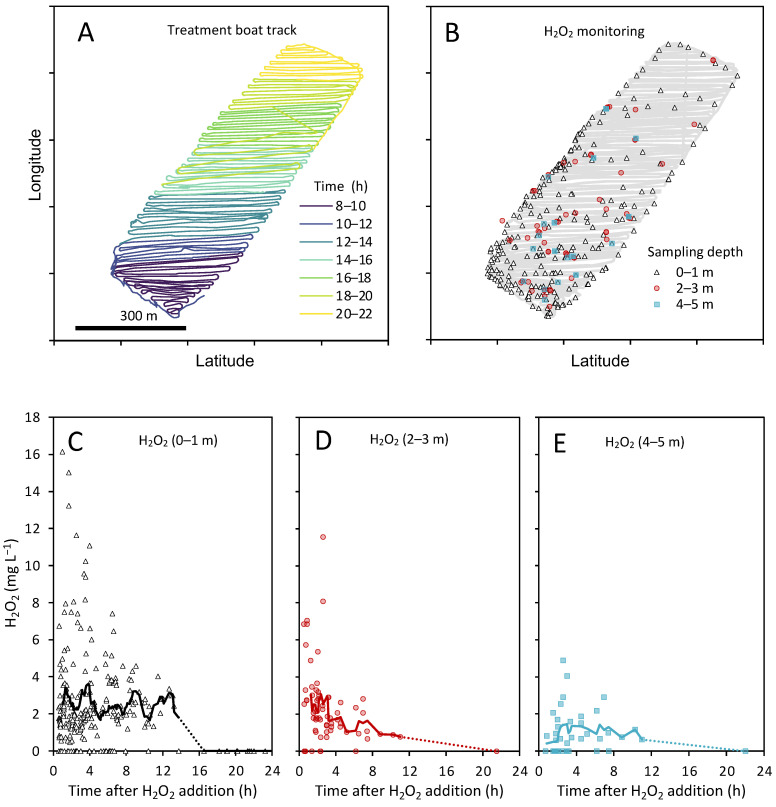
H_2_O_2_ treatment of the lake. The graph shows (**A**) the boat track during the treatment in August, (**B**) the sampling locations and the sampling depth where H_2_O_2_ concentrations were monitored during the treatment. (**C**–**E**) H_2_O_2_ concentrations measured at different time points after the treatment boat had passed the sampling locations, (**C**) at 0–1-m depth, (**D**) at 2–3-m depth and (**E**) at the 4–5-m depth. Colors in panel (**A**) indicate the time of day during which a certain section of the lake was treated; different symbols in panel (**B**) indicate the sampling depth; lines in panels (**C**–**E**) are moving averages with a window size of 60 min.

In 2018, lake Oosterduinse Meer was treated twice with H_2_O_2_; the first treatment took place on 19 June, the second treatment—on 7 August (Figure 1B). During each treatment, H_2_O_2_ was applied to the top 5 m of the entire lake using a specialized boat (Figure 1C), carefully adding 4.2 mL of a 598 g L^−1^ H_2_O_2_ (50% H_2_O_2_ (*w*/*w*)) stock per 1 m^3^ of the lake water to treat the lake with a target concentration of ~2.5 mg L^−1^ H_2_O_2_. During the June treatment, the boat first treated the shallower waters along the shore of the lake, then went to the lake center, treating the sampling location by spiraling three times around it, before covering the rest of the lake from south to north with 75 diagonal lanes (Figure S1A). The average distance between the lanes was 13.3 m and the net treatment time was 7.3 h. During the August treatment, the boat navigated the lake from south to north with 91 diagonal lanes, while the shallower parts along the shore were treated in between (Figure 2). The average distance between the lanes was 10.9 m and the net treatment time was 9.2 h.

During the treatments, continuous measurements of temperature, O_2_ saturation, pH and light intensity were collected using a water column (surface to the bottom) from the center of the lake using a Hydrolab Datasonde 5 (OTT Messtechnik GmbH and Co., Kempten, Germany). Weather data from the weather station Schiphol located ~20 km east of the lake were provided by the Royal Netherlands Meteorological Institute (KNMI) (www.knmi.nl; accessed on 10 January 2020).

Samples for DNA extraction, phytoplankton identification and quantification, analyses of nutrient concentrations and bacterial enumeration were taken from the 0-m and 5-m depth at the sampling location in the middle of the lake. The samples from the 5-m depth were pumped up with a 1.4-bar water pump, type 088 (Barwig Wasserversorgung, Bad Karlshafen, Germany), connected to a 5-m-long tube. One day before the treatment (t = −24 h), seven biological replicates were sampled from the 0-m depth, six biological replicates—from the 5-m depth. For all the other time points, at 0 h (just before the treatment, only for phytoplankton and nutrient analysis), 5 h, 24 h, 48 h and 96 h after the treatment, four biological replicates were taken from both depths. All the samples were collected in individual 5-L transparent plastic bags (DaklaPack, Lelystad, The Netherlands) and immediately processed on the shore.

For DNA extraction, a 1-L subsample was filtered instantly through a 5-µm polycarbonate Cyclopore filter (Whatman GmbH, Dassel, Germany) on a Nalgene™ filtration unit (ThermoScientific, Waltham, MA, USA) to remove most filamentous cyanobacteria, bigger eukaryotic organisms and particles. The filtrate containing planktonic bacteria that were not attached to bigger particles was collected and filtered again over a 0.2-µm polycarbonate Cyclopore filter (Whatman GmbH, Dassel, Germany). This filter was then carefully folded, placed into a 1.5-mL screw cap tube and immediately snap frozen in a CX100 dry shipper (Taylor-Wharton/Worthington Industries, Columbus, OH, USA) at −190 °C and later stored at −80 °C until analysis. Only 0.2-µm polycarbonate Cyclopore filters were used for DNA extraction.

For phytoplankton analysis, 30-mL subsamples were taken from three of the biological replicates, fixed with 600 µL acidic Lugol’s iodine and immediately stored at 4 °C until further analysis.

For bacterial enumeration using flow cytometry, a 1.5-mL subsample was taken from each replicate, fixed with glutaraldehyde (0.5% (*v*/*v*) final concentration prepared from a 25% (*v*/*v*) EM grade stock solution; VWR, Amsterdam, The Netherlands) at 4 °C for 15 min, after which it was snap frozen and later stored at −80 °C until analysis.

For the quantification of dissolved inorganic nutrients, 55-mL subsamples from each biological replicate were filtered over GF/C filters on a Millipore 1225 Sampling Manifold (Merck KGaA, Darmstadt, Germany). Subsequently, 15 mL of each filtrate were frozen and stored at −20 °C for nutrient analysis.

### 2.2. Incubation Experiments

Each lake treatment was accompanied by in situ incubation experiments with two H_2_O_2_ concentrations and a control. In contrast to an entire lake treatment aimed to provide valuable insight to a real-life scenario, these small-scale lake incubations allowed replication of different H_2_O_2_ treatments including controls without H_2_O_2_, which enables a more controlled (but less “realistic”) study of the effect of H_2_O_2_ on microbial communities.

One day before a lake treatment (t = −24 h), 47 transparent 5-L food-grade plastic bags (DaklaPack, Lelystad, The Netherlands) were filled with lake water from 0 m. These incubation bags were divided into nine groups of four bags each (to be sampled at t = 24, t = 48 and t = 96 h), one group of three bags (t = 0 h) and one group of eight “refill bags” (Figure 1D). All the incubation bags were attached to a raft at the sampling location at the lake center. A weight was attached to each group of bags, holding them submerged below the water at a constant depth of 1 m.

On the treatment day, at the time that the treatment boat passed the sampling location, 12 incubation bags received 10 mL of Milli-Q water to serve as controls (0 mg L^−1^ H_2_O_2_), 12 incubation bags received 10 mL of a diluted H_2_O_2_ stock solution at a final concentration of 2.5 mg L^−1^ H_2_O_2_ and another 12 incubation bags received 10 mL of an H_2_O_2_ stock solution at a final concentration of 10 mg L^−1^ H_2_O_2_ (Figure 1D). After each addition of H_2_O_2_, water of the eight “refill bags” was used to completely fill up all the treated incubation bags to their total volume so that no air remained inside. At last, the three incubation bags of the t = 0 group were treated with Milli-Q water, 2.5 and 10 mg L^−1^ H_2_O_2_, respectively, directly brought to the shore and sampled 10 min later. From each of these three incubation bags (t = 0), three individual subsamples were taken to measure the added H_2_O_2_ as well as to monitor phytoplankton and quantify dissolved inorganic nutrient concentrations. At each consecutive time point (t = 24, 48 and 96 h), four incubation bags from each of the three treatments (0 mg L^−1^, 2.5 mg L^−1^ and 10 mg L^−1^ H_2_O_2_) were removed from the raft and brought to the shore for immediate analysis (Figure 1D). All four incubation bags from each of the three experimental treatments were sampled for DNA extraction, nutrient analysis and bacterial abundances, while three incubation bags from each experimental treatment were sampled for phytoplankton analysis. The samples were handled and stored in the same way as described above for the field sampling.

### 2.3. H_2_O_2_ Measurements

The H_2_O_2_ concentration was frequently measured at several locations across the lake throughout the treatment day using peroxide quantification strips (sensitivity range: 0.5–25 mg L^−1^, Quantofix^®^, Macherey-Nagel GmbH and Co., KG; Dueren, Germany) and a mobile strip reader (Quantofix^®^, Macherey-Nagel GmbH and Co., KG). Only those measurements that were taken at least 30 min after the boat had treated the monitored location were considered. Time and GPS coordinates of the boat and each monitored location were used to calculate the time difference between the time of sampling and the time the boat passed nearest (and latest) by the sampling location. The H_2_O_2_ concentrations from the raft location were additionally measured regularly with a more sensitive colorimetric assay (*p*-nitrophenylboronic acid) according to Lu et al. [57] and Piel et al. [38]. These measurements were also used to calibrate the strip measurements. In short, 55-mL samples from each of the sampling depths were filtered through GF/C glass fiber filters (Whatman GmbH, Dassel, Germany), and 100 µL of the filtrate were immediately mixed with 100 μL of 2 mM *p*-nitrophenylboronic acid reagent (Merck KGaA, Darmstadt, Germany) in a 96-well plate. After incubating the assay for 30–45 min in almost complete darkness, the absorption of *p*-nitrophenolate was measured at its absorption peak (405 nm) using a plate reader (Multiscan FC type 357, ThermoScientific, Waltham, MA, USA). Each sample was analyzed in triplicates with each replicate consisting of three technical replicates. A 33% (*w*/*w*) stock solution (VWR, Amsterdam, The Netherlands) was used to make a calibration curve (in the range of 0.01–10 mg L^−1^ H_2_O_2_) that was included on each 96-well plate. Since this assay is extremely sensitive to sunlight and cannot be easily implemented in the field, it was necessary to perform all the steps of this analysis indoors in the dark. The measurements of the strips and the colorimetric assay showed a close correlation (R^2^ = 0.93).

### 2.4. Quantification of Dissolved Inorganic Nutrients

The filtrates for nutrient analysis (see details above) were thawed and subsequently filtered over 0.2-μm pore size 25-mm Whatman polycarbonate membrane filters (GE Healthcare, Buckinghamshire, UK). Concentrations of dissolved ammonia, nitrates, nitrites and phosphates were measured using a San++ Automated Wet Chemistry Analyzer (Skalar Analytical B.V., Breda, The Netherlands) at a detection limit of 0.10 µmol L^−1^, 0.02 µmol L^−1^, 0.03 µmol L^−1^ and 0.03 µmol L^−1^, respectively.

### 2.5. Microscopic Analysis of Phytoplankton

Phytoplankton in a 1-mL subsample of the sample fixed with Lugol’s iodine was identified to the genus or species level using an inverted microscope (Zeiss IM35, Oberkochen, Germany) and quantified following the Utermöhl method [58] using a counting chamber. Biovolumes of the phytoplankton were calculated from cell numbers, measured cell sizes and cellular geometry according to [59].

### 2.6. Flow Cytometric Analysis of Prokaryotes

Single-celled prokaryotes (bacteria, archaea) were quantified using a Becton Dickinson FACSCalibur flow cytometer (BD Biosciences, San Jose, CA, USA) according to the protocol suggested by Marie et al. [60]. The thawed samples were diluted in 0.2 µm filtered (FP 30/0.2 CA-S Whatman, Dasser, Germany) 10:1 Tris–EDTA buffer (pH 8), stained with the nucleic acid-specific green fluorescent dye SYBR Green I at a final concentration of 1 × 10^−4^ commercial stock (Invitrogen Molecular Probes, Eugene, OR, USA) for 15 min in the dark at room temperature, followed by flow cytometric analysis with the trigger set on green fluorescence. The microbial cells were discriminated by plotting green fluorescence against the side scatter using Flowing Software (version 2.5.1; www.flowingsoftware.com; accessed on 7 January 2020).

### 2.7. DNA Extraction

DNA was extracted using a DNeasy^®^ PowerSoil^®^ extraction kit (Qiagen, Hilde, Germany) according to the manufacturer’s guidelines. The extracted DNA concentrations were quantified using a Qubit 2.0 fluorometer (Invitrogen, Carlsbad, CA, USA) and diluted to ~25 ng µL^−1^ before drying in a CentriVap Concentrator (Labconco, Kansas City, MO, USA). Dried DNA was then shipped at room temperature for sequencing.

### 2.8. 16S rRNA Gene Amplicon Sequencing and Data Analysis

First, the lake samples were used to compare the suitability of different primer pairs for 16S rRNA gene amplicon sequencing. With general primer pairs covering the V3–V5 region (i.e., 515F–Y/926R), however, cyanobacteria comprised more than 80% of the reads per sample, whereas the number of reads of most other bacteria in the microbial community was low. Since cyanobacteria were already quantified by microscopy, we therefore decided to use the primer pair 799F (5′-AACMGGATTAGATACCCKG-3′) and 1193R (5′-ACGTCATCCCCACCTTCC3′) [61,62], which targets the V5–V7 region of the 16S rRNA gene but specifically avoids amplification of chloroplast DNA and cyanobacteria. The main advantage of this approach is that high resolution of the microbial community can be achieved because the data are not cluttered by large numbers of cyanobacterial reads. A 30-cycle PCR was performed by MR DNA (Shallowater, TX, USA) with added barcodes to the forward primer using a HotStarTaq Plus Master Mix Kit (Qiagen, Germantown, MD, USA) under the following conditions: 94 °C for 3 min followed by 30 cycles of 94 °C for 30 s, 53 °C for 40 s and 72 °C for 1 min, with the final elongation step at 72 °C for 5 min. PCR products were checked on a 2% agarose gel to determine amplification success. The samples were then pooled in five equal pools each with equal proportions of molecular weight and DNA concentrations and purified with calibrated AMPure XP beads. All the five pools were then used to prepare the Illumina DNA libraries. Each pool was sequenced on a separate run on an Illumina MiSeq at MR DNA (Shallowater, TX, USA) according to the manufacturer’s guidelines. In addition, three samples were added to each of the five individual runs to verify that the run-to-run variation was minimal (Figure S2). Furthermore, four replicates of the so-called “mock community,” a mixture of 10 well-characterized bacterial strains in equal amounts (MSA-1000™, ATCC^®^, Manassas, VA, USA), were added to confirm classification accuracy and check for possible primer bias. Analysis of the mock community results with the evaluate-composition command of the q2-quality control plugin of QIIME2 version 2019.4 [63] indicated that at the genus level, the taxon detection rate was 100% and that the relative abundances of the observed taxa were close to the expected levels.

The sequence data for this study were deposited in the European Nucleotide Archive (ENA) at the EMBL-EBI under accession number PRJEB44985 (https://www.ebi.ac.uk/ena/browser/view/PRJEB44985; accessed on 6 April 2021) and the corresponding metadata are summarized in Table S1.

Raw sequences were transformed with the FASTQ processer of MR DNA (Shallowater, TX, USA) before analysis with QIIME2 (versions 2019.4 and 2019.10) [63]. The sequences were demultiplexed [64,65], then joined and quality-trimmed during DADA2 denoising [66] to a quality score of at least 20 and with an overlap of at least 20 nucleotides. Feature tables of amplicon sequencing variants (ASVs) and representative sequences (rep-seqs) tables of all the five runs were subsequently merged using the q2-feature-table plugin. Taxonomic classification was performed with the q2-feature-classifier plugin [67] by first training the machine-learning classifier on the sequences generated by the primer (799F/1193R) using the fit-classifier-naive-bayes command [68]. The subsequent classification was performed using the classify-sklearn command and 99% SILVA database version 132 as the reference [69,70]. A phylogenetic tree (SEPP) was generated using the q2-fragment-insertion plugin [71] to allow for phylogenetic diversity analyses such as UniFrac distances and Faith’s phylogenetic diversity (PD).

Alpha diversity analysis was performed on rarefied but unfiltered feature tables. To allow for comparisons of alpha diversity between all the treatments and conditions of this study, all the samples were rarefied to the same sampling depth of 22,554 using the rarefy command in the feature-table plugin [72]. Chao-1 index and Faith’s PD were generated using the q2-diversity plugin. Pairwise Kruskal–Wallis tests were used to compare the indices of different time points.

Beta diversity was quantified as Bray–Curtis dissimilarity and as UniFrac distances. Mild low-frequency filtration was applied to all the samples to remove rare or potentially faulty features (i.e., features with less than four reads were removed). With the core-metrics command of the q2-diversity plugin, all the lake samples were rarefied to a minimal sampling depth of 22,554, all the samples of the lake incubations (including the t = −24 samples)—to a minimal sampling depth of 23,593 before calculating Bray–Curtis dissimilarity and unweighted UniFrac distance matrices [73,74,75,76,77,78]. Principal coordinates analysis (PCoA) plots visualizing the beta diversity matrices were generated in QIIME2 (versions 2019.4 and 2019.10). Subsequently, a pairwise PERMANOVA was used to calculate significant differences between clusters in the same PCoA plot [79].

Relative abundance plots were generated for bacterial abundances in the lake (at the 0-m and 5-m depth) and in the incubation experiments for both the June and August treatments using the 50 most abundant taxa at the order level (covering more than 99% of the relative abundances) and the 25 most abundant taxa at the genus level. The graphs were generated with the original unrarefied feature tables as rarefaction is not required with centered log ratio (clr) transformation [80]. Since clr transformation is sensitive to sparse data with many zero values, a prior filtration step was included to filter out all the rare features < 25 reads and presence in less than four samples. This filtration step resulted in a 58.38 ± 2.27% decrease in the number of features, but only in a 0.55 ± 0.15% decrease in the total read counts. Despite the strong decrease in features, the Mantel test between unfiltered and filtered dissimilarity/distance matrixes confirmed significant correlation. Sequence counts were clr-transformed with the aldex.clr function of the package ALDEx2 (version 1.18.0) [81,82] in R (version 3.6.2) and the median of 128 Monte Carlo Dirichlet instances was extracted. The dot plot was generated using the scales package (version 1.1.0) in R (version 3.6.2) and a slightly adjusted DotPlot function according to Guevara Campoverde et al. [83].

Differential abundance between the control incubations (0 mg L^−1^ H_2_O_2_) and the treated incubations (2.5 mg L^−1^ and 10 mg L^−1^ H_2_O_2_) 24 h and 96 h after addition of H_2_O_2_ was calculated using filtered clr-transformed feature tables that were collapsed at the order and genus level and the aldex.ttest and aldex.effect functions [81,82,84]. The effect size as calculated in ALDEx2 (0.7 × Cohen’s *d*) is shown in a heat map for each comparison with a significant Welch’s *t*-test (*p* < 0.05 after correction for multiple hypothesis testing according to Benjamini and Hochberg [85]). Heat maps of the effect size of differentially relative abundance orders was generated using the gplots (version 3.0.3) package in R (version 3.6.2).

To predict ecological functions of bacteria during the treatments, the samples were analyzed with PICRUSt2 [86] and Tax4Fun2 [87]. Techniques and databases for functional predictions are still in development, and PICRUSt2 and Tax4Fun2 follow different methods and make use of different reference databases. Therefore, we analyzed our data with both pipelines and compared their predictions to assess the robustness of the results. The predicted KEGG orthologs of both pipelines were corrected by the 16S gene copy number [86,87]. The selected KEGG orthologs classified as glycoside hydrolases according to the Carbohydrate Active Enzymes database ([88]; http://www.cazy.org; accessed on 12 January 2020) as well as the orthologs involved in the anti-ROS activity or belonging to fundamental nutrient cycling pathways according to the KEGG database [89,90,91] were clustered and analyzed further. The PICRUSt2 predicted the KEGG orthologs were clr-transformed, and the differential relative abundances between the experimental treatments and the control of the lake incubations were calculated using ALDEx2 as mentioned above. The Tax4Fun2 prediction output of the KEGG orthologs is given as percentage values of relative abundances and could not be clr-transformed as the PICRUSt2 data. The output values were therefore log_2_-transformed and the Wilcoxon test with *p*-values corrected for multiple hypothesis testing [85] was used to calculate the differential relative abundances of the KEGG orthologs between the experimental treatments and the control. Dot plots and heat maps indicating significant increases or decreases in the relative abundances were generated using the gplots (version 3.0.3) package in R (version 3.6.2).

## 3. Results

The results of the two H_2_O_2_ treatments of the lake were quite comparable. For conciseness, we present only the August treatment in detail here, and highlight important differences with the June treatment (Supplementary Materials) at the end of Section 3. From now on, we refer to the August treatment as “the treatment” unless specified otherwise.

### 3.1. H_2_O_2_ Concentrations during the Treatment

The lake treatment aimed to obtain the target H_2_O_2_ concentration of 2.5 mg L^−1^ across the top 5 m of the entire lake by careful injection of H_2_O_2_ from a specially designed boat (Figure 1C). Measurements in the surface water (0–1-m depth) at various locations across the entire lake (Figure 2B) showed relatively stable average concentrations between 2 and 3 mg L^−1^ H_2_O_2_ for up to ~13 h after the treatment boat had passed (Figure 2C). Deeper in the lake (2–3- and 4–5-m depth), the average concentrations varied between 0.7 and 3 mg L^−1^ H_2_O_2_ during this time period (Figure 2D,E). One day later (at t > 20 h), the added H_2_O_2_ was degraded to <0.01 mg L^−1^.

In the incubation experiments, the first H_2_O_2_ measurement was about 10 min after the H_2_O_2_ addition, displaying a rapid decline to 1.6 ± 0.1 mg L^−1^ H_2_O_2_ in the 2.5 mg L^−1^ treatment and to 8.5 ± 0.6 mg L^−1^ H_2_O_2_ in the 10 mg L^−1^ treatment. The added H_2_O_2_ was degraded to <0.01 mg L^−1^ in all the incubation experiments after 24 h.

### 3.2. Environmental Data during the Treatment

The treatment day was characterized by very warm weather (maximum air temperature of 33.7 °C) accompanied by vertical microstratification of the lake temperature and supersaturated oxygen concentrations in the surface layer of the lake (Figure S3, Table S2). The subsequent cooler weather resulted in a more homogeneous temperature distribution over the upper 4 m of the epilimnion on the day after the treatment followed by a further deepening of the thermocline to ~6-m depth during the next few days (Figure S3B). Dissolved oxygen in the surface layer declined in the days after the H_2_O_2_ treatment but penetrated deeper into the water column as the anoxic hypolimnion gradually moved from the 4-m depth prior to the treatment to > 6-m depth at day 4 after the treatment (Figure S3D). The pH in the surface layer declined from 9.5 before the treatment to 8.7 in the days after the treatment (Figure S3F). The lake was very turbid prior to the treatment, with a euphotic depth (defined as the depth receiving 1% of the surface irradiance) of ~0.9 m. Within 5 h after the start of the treatment, sunlight penetrated deeper into the water column, and after 4 days, the euphotic zone extended to ~2.6-m depth (Figure S3H).

The NH_4_^+^ and PO_4_^3−^ concentrations increased after the H_2_O_2_ treatment of the entire lake, with a particularly pronounced increase of NH_4_^+^ at the 0-m depth (whereas the NO_3_^−^ and NO_2_^−^ concentrations remained low; Figure S4). A similar strong increase of the NH_4_^+^ concentration was also found in the lake incubations treated with 2.5 and 10 mg L^−1^ H_2_O_2_, whereas the NO_3_^−^, NO_2_^−^ and PO_4_^3−^ concentrations in the lake incubations remained largely unaltered after the treatment (Figure S4).

### 3.3. Effects of H_2_O_2_ on Phytoplankton

**Figure 3:**
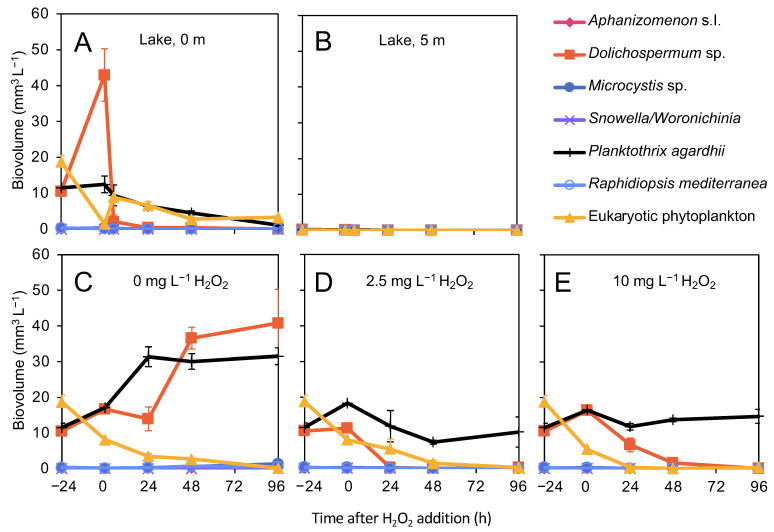
Phytoplankton composition during the H_2_O_2_ treatment in August. (**A**,**B**) Biovolume of the most abundant cyanobacterial taxa and of the total eukaryotic phytoplankton at (**A**) the 0-m and (**B**) 5-m depth during the H_2_O_2_ treatment of the lake in August. (**C**–**E**) Biovolumes in the incubation experiments treated with (**C**) 0 mg L^−1^, (**D**) 2.5 mg L^−1^ and € 10 mg L^−1^ of H_2_O_2_. The data show the mean (±SD) of *n* = three biological replicates per time point.

Prior to the lake treatment, *Planktothrix agardhii* and *Dolichospermum* sp. Were the dominant cyanobacteria at the surface of the lake with biovolumes of 11.5 ± 1.2 mm^3^ L^−1^ and 10.5 ± 1.1 mm^3^ L^−1^, respectively (Figure 3A). The biovolumes of other cyanobacteria, including *Microcystis* and *Aphanizomenon*, were <0.1 mm^3^ L^−1^. Eukaryotic phytoplankton (almost exclusively consisting of the dinoflagellate *Ceratium* spp.) were also highly abundant in the surface layer before the treatment with a total biovolume of 18.7 ± 1.8 mm^3^ L^−1^. Cyanobacteria and eukaryotic phytoplankton were hardly present at the 5-m depth (Figure 3B). As soon as 5 h after the lake treatment, the biovolume of the surface-dwelling *Dolichospermum* sp. Was reduced by 95%, and it completely disappeared in the subsequent days. The biovolume of *P. agardhii* declined steadily, but at a much lower rate than the biovolume of *Dolichospermum* sp. The biovolume of eukaryotic phytoplankton also decreased after the treatment before stabilizing between 48 and 96 h after the treatment (Figure 3A).

In the control of the incubation experiments (without H_2_O_2_ addition), the biovolume of the cyanobacteria strongly increased while the biovolume of eukaryotes (again, mostly *Ceratium* spp.) declined. In the H_2_O_2_-treated incubations, the cyanobacterium *Dolichospermum* sp. and the eukaryotic phytoplankton disappeared almost completely within 24 to 48 h, whereas the biovolume of *P. agardhii* remained stable throughout the entire experiment (Figure 3C–E).

### 3.4. Bacterial Abundances

**Figure 4:**
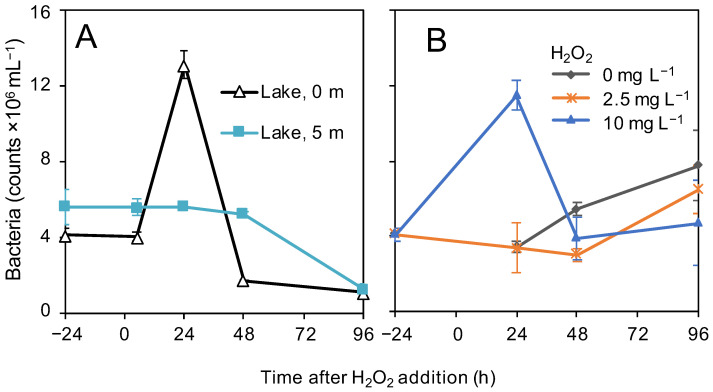
Total bacterial abundances during the H_2_O_2_ treatment in August. Total bacterial abundances as determined with flow cytometry during (**A**) the lake treatment and (**B**) the incubation experiments in August. The data show the mean (±SD) based on *n* = seven biological replicates for t = −24 h at 0 m (**A**), *n* = six biological replicates for t = −24 h at 5 m (**B**) and *n* = four biological replicates for all the other time points.

In the surface layer (0 m) of the lake, bacterial abundances peaked 24 h after the addition of H_2_O_2_, after which they declined again. In contrast, at the 5-m depth, bacterial abundances remained stable for the first 48 h, followed by a decline to values similar to those at 0 m (Figure 4A). In the control and the 2.5 mg L^−1^ H_2_O_2_-treated incubations, bacterial abundances slowly increased towards the end of the experiment. In the 10 mg L^−1^ H_2_O_2_-treated incubations, bacterial numbers increased in the first 24 h before returning to the initial numbers (Figure 4B).

### 3.5. Microbial Community Analysis

#### 3.5.1. Microbial Community Composition in the Lake

**Figure 5:**
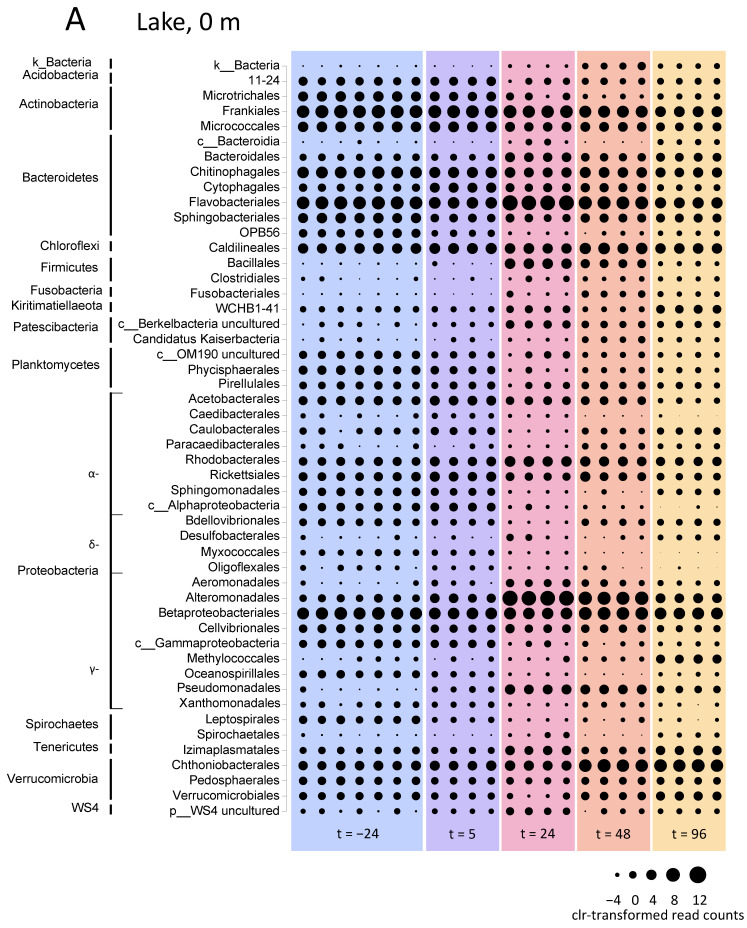

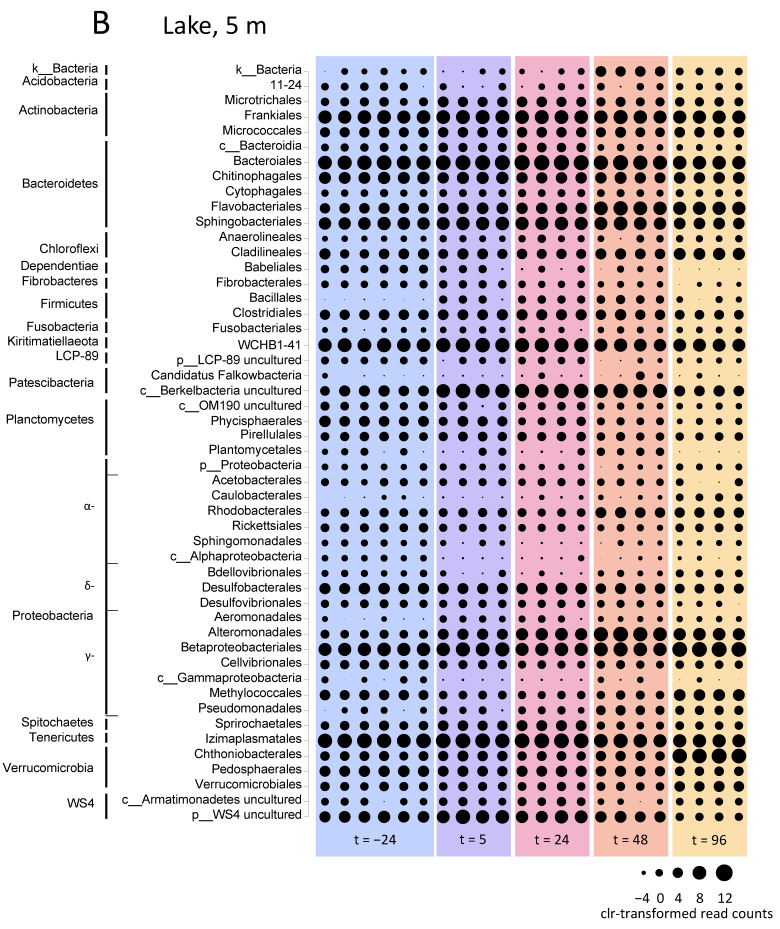
Microbial community composition during the lake treatment in August. Relative abundance shown as clr-transformed read counts at the phylum and order levels at (**A**) the 0-m and (**B**) 5-m depth during the lake treatment in August. Columns of the same color represent biological replicates at the same time point; columns of different colors represent different time points; “k__”, “p__” and “c__” indicate that the maximum classification of these taxa is at the kingdom, phylum and class level, respectively.

The microbial community composition showed clear differences between the oxygen-rich surface layer and the oxygen-depleted waters at the 5-m depth already prior to the H_2_O_2_ treatment of the lake (Figure 5). Although the orders Frankiales and Betaproteobacteriales had high relative abundances at both depths, Flavobacteriales, for example, showed high relative abundances in the surface layer, whereas Bacteroidales, Sphingobacteriales, WCHB1-41 and Izimaplasmatales reached high relative abundances at the 5-m depth (Figure 5).

The bacterial taxa that showed a strong increase in the relative abundance after the H_2_O_2_ treatment in the surface layer of the lake included the orders Bacteroidales, Flavobacteriales, Bacillales, Alteromonadales, Methylococcales, Pseudomonadales, Izimaplasmatales and Chthoniobacterales (Figure 5A). In particular, the genera *Rheinheimera* (Alteromonadales) and *Flavobacterium* (Flavobacteriales) increased from 0.2% and 23% at 24 h before H_2_O_2_ addition to 44% and 45% of the microbial community at 24 h after H_2_O_2_ addition (Figure S5A; Table S3). Their relative abundances fell back again during the subsequent days, however, to 2% for *Rheinheimera* and 10% for *Flavobacterium* at 96 h after H_2_O_2_ addition. Conversely, the taxa that showed a decrease in the relative abundance after the H_2_O_2_ treatment included the orders Microtrichales, OPB56, Caedibacterales, unclassified Alphaproteobacteria, Myxococcales, Oligoflexales and Leptospirales (Figure 5A). 

The relative abundances at the 5-m depth were somewhat more stable. The taxa that increased in the relative abundance at the 5-m depth after the H_2_O_2_ treatment included Flavobacteriales, uncultured Berkelbacteria, Rhodobacterales, Alteromonadales (again, especially *Rheinheimera*), Methylococcales, Pseudomonadales and Chthoniobacterales, while the taxa that decreased in the relative abundance included Babeliales and Phycisphaerales (Figure 5B; Figure S5B).

#### 3.5.2. Microbial Community Composition in the Incubation Experiments

**Figure 6:**
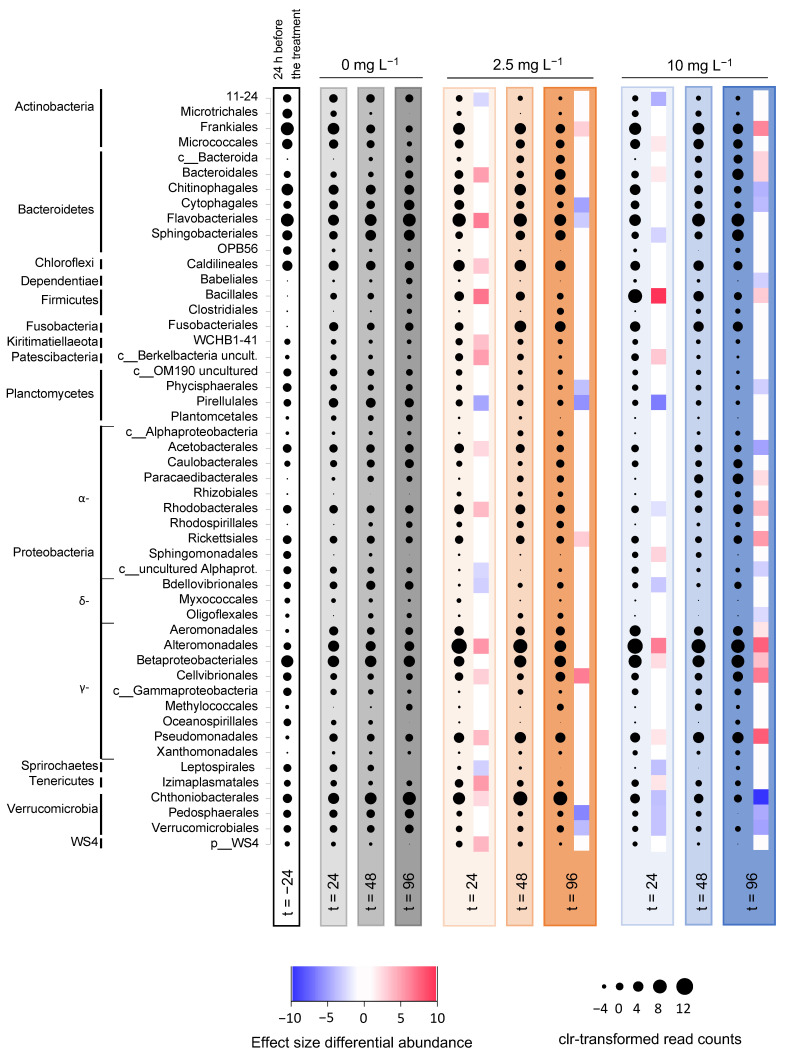
Microbial community composition during the incubation experiments in August. The graph shows the relative abundances as clr-transformed read counts of bacteria at the order level for each of the time points during the August treatment. The first column represents the average relative abundances in the lake prior to the incubation experiments (t = −24 h, with *n* = seven biological replicates). The other columns represent the relative abundances in the incubation experiments at three subsequent time points (t = 24, 48 and 96 h, with *n* = four biological replicates per time point). Gray columns represent the control incubations (0 mg L^−1^ H_2_O_2_), orange columns—the 2.5 mg L^−1^ H_2_O_2_-treated incubations, blue columns—the 10 mg L^−1^ H_2_O_2_-treated incubations. Differential abundances of orders between the treated and control incubations at t = 24 h and t = 96 h were calculated using ALDEx2. Effect sizes of the statistical test (0.7 × Cohen’s *d*) are shown in the heat map next to the t = 24 and t = 96 columns for all the significant comparisons; blue indicates a significant decrease in the relative abundance while red indicates a significant increase in the relative abundance in comparison to the control; “p__” and “c__” indicate that the maximum classification of these taxa is at the phylum and class level, respectively.

The incubation experiments showed a strong initial increase in the relative abundance of Alteromonadales, and especially the genus *Rheinheimera*, in response to the H_2_O_2_ treatment (Figure 6; Figure S6). At t = 24 h, *Rheinheimera* increased to 73% and 62% of the microbial community in the incubations treated with 2.5 and 10 mg L^−1^ H_2_O_2_, respectively, whereas it comprised only 12% of the microbial community in the control without H_2_O_2_ (Table S3). During the subsequent days, at t = 96 h after H_2_O_2_ addition, the relative abundance of *Rheinheimera* declined again to 8% and 34% of the microbial community in the treatments with 2.5 and 10 mg L^−1^ H_2_O_2_, respectively. The other taxa with a significantly higher relative abundance after the addition of 2.5 mg L^−1^ H_2_O_2_ than in the control included the orders Frankiales, Rickettsiales and Cellvibrionales, while the taxa with a significantly lower relative abundance included Cytophagales, Flavobacteriales, Phycisphaerales, Pirellulales, Pedosphaerales and Verrucomicrobiales (Figure 6). The other taxa with a significantly higher relative abundance after the addition of 10 mg L^−1^ H_2_O_2_ included Frankiales, unclassified Bacteroidia, Bacteroidales, Bacillales, Paracaedibacterales, Rhodobacterales, Rickettsiales, Aeromonadales, Betaproteobacteriales, Cellvibrionales and Pseudomonadales, while the taxa with a significantly lower relative abundance included Chitinophagales, Cytophagales, Babeliales, Phycisphaerales, Acetobacterales, uncultured Alphaproteobacteria, Oligoflexales, Chthoniobacterales, Pedosphaerales and Verrucomicrobiales (Figure 6).

#### 3.5.3. Microbial Diversity

**Figure 7:**
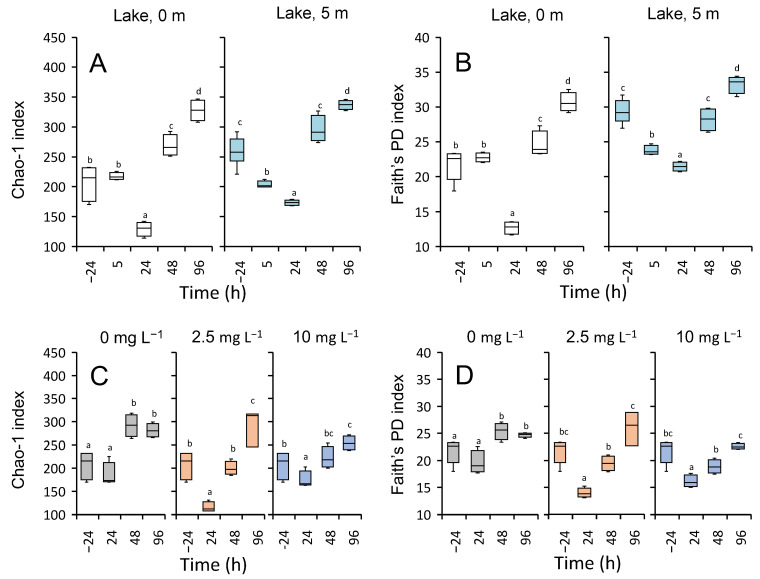
Alpha diversity of the microbial community during the H_2_O_2_ treatment in August. (**A**,**C**) The Chao-1 index (estimated species richness), and (**B**,**D**) Faith’s phylogenetic diversity during (**A**,**B**) the lake treatment and (**C**,**D**) the incubation experiments in August. The color of the box plots indicates (**A**,**B**) the sampling depth in the lake or (**C**,**D**) the different H_2_O_2_ treatment concentrations of the incubations. The horizontal lines within the box plots represent the median, the boxes—the 25% quartile and the 75% quartile, error bars—the minimum values and the maximum values. The first box plot in each panel represents the alpha diversity in the lake prior to the incubation experiments (t = −24 h, with *n* = seven biological replicates). Box plots of all the subsequent time points are based on *n* = four biological replicates. Box plots with different letters indicate significant differences between time points according to pairwise Kruskal–Wallis tests.

**Figure 8:**
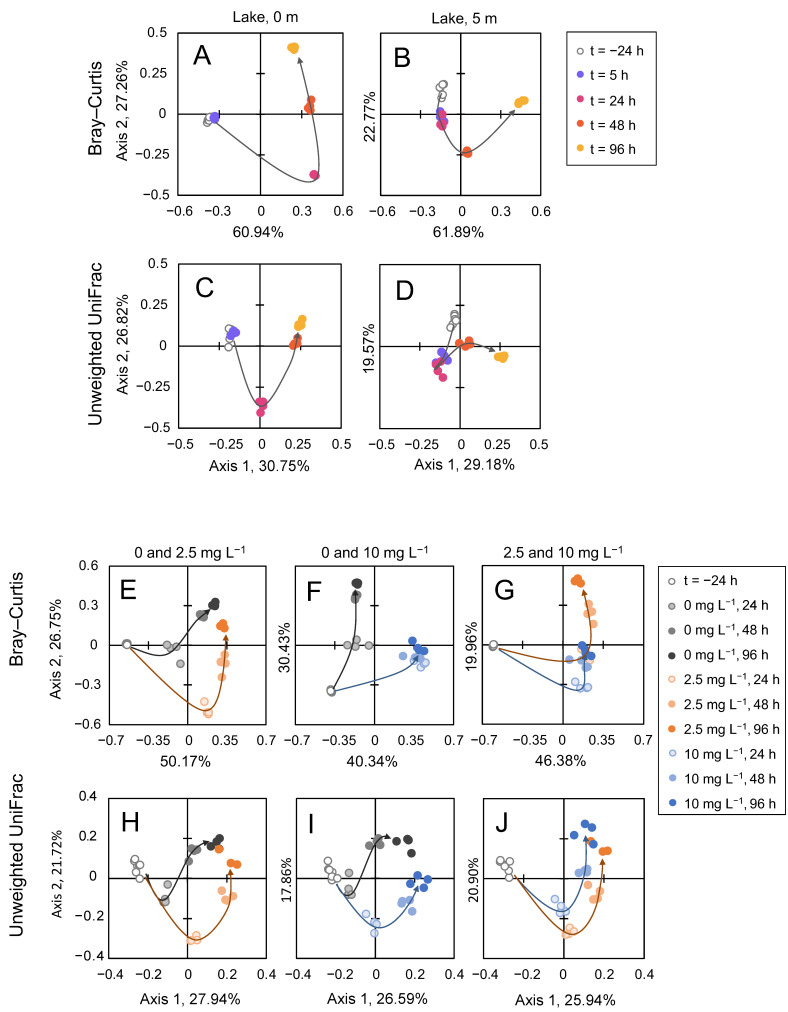
Beta diversity of the microbial community during the H_2_O_2_ treatment in August. PCoA plots of beta diversity of the microbial community during (**A**–**D**) the lake treatment and (**E**–**J**) the incubation experiments in August. Beta diversity in (**A**,**B**) and (**E**–**G**) is based on the Bray–Curtis dissimilarity matrix, in (**C**,**D**) and (**H**–**J**)—on the unweighted UniFrac distance matrix. Each symbol in the graph represents one sample; all samples with symbols of the same color are biological replicates. Gray open circles represent samples in the lake prior to the H_2_O_2_ treatment (i.e., the starting community at t = −24 h). Arrows visualize the trajectories of the bacterial communities throughout the experiment. Pairwise PERMANOVAs revealed significant differences between all clusters within the same panel.

The two alpha diversity indices, species richness (Chao-1) and phylogenetic diversity (Faith’s PD), decreased significantly after the addition of H_2_O_2_ at both depths in the lake. The initial decline in alpha diversity, which reached the minimum at 24 h after the treatment, was followed by a strong and significant recovery after 48 h that even further increased after 96 h, surpassing the diversity observed before the lake treatment (Figure 7A,B). Recovery of the microbial communities after the lake treatment was also indicated by the beta diversity analysis using Bray–Curtis dissimilarity matrices and unweighted UniFrac distance matrices. Strong shifts in the microbial communities after the treatment were followed by V-shaped recovery patterns along axis 2 of the PCoA plots both for Bray–Curtis dissimilarity (Figure 8A,B) and unweighted UniFrac distances (Figure 8C,D).

Diversity indices of the incubation bags showed very similar responses to H_2_O_2_ addition as in the lake. The Chao-1 index and Faith’s PD declined significantly 24 h after the treatment with 2.5 mg L^−1^ and 10 mg L^−1^ H_2_O_2_, followed by a strong and significant recovery until the end of the experiment (Figure 7C,D). The beta diversity trajectories of the treated and control communities initially diverged but then converged during the subsequent 48–96 h. Convergence towards the community composition of the control is indicative of a strong recovery of the microbial communities. When analyzed using Bray–Curtis dissimilarity, a metric that takes relative abundances into account and therefore focuses on the more abundant features, the 2.5 mg L^−1^ H_2_O_2_-treated communities showed a clear recovery whereas the trajectories suggested only a partial recovery for the 10 mg L^−1^ H_2_O_2_ treatment (Figure 8E–G). When analyzed with unweighted UniFrac distances, a metric that focuses on the rarer features, a strong recovery was observed after the treatment with both H_2_O_2_ concentrations (Figure 8H–J).

#### 3.5.4. Functional Prediction

**Figure 9:**
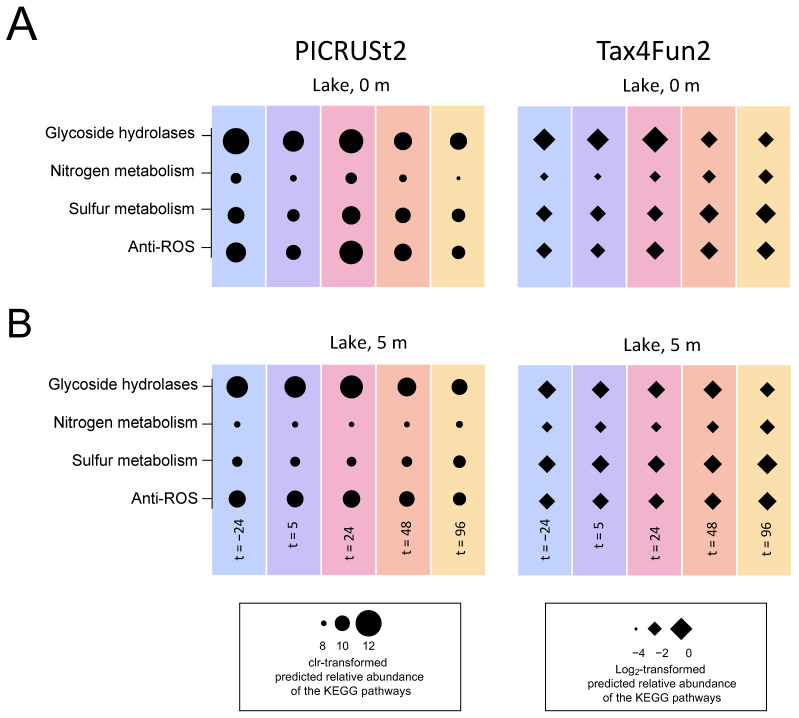
Functional prediction of the microbial community during the lake treatment in August. The graphs show predicted relative abundances of the selected KEGG orthologs or pathways for glycoside hydrolases, nitrogen and sulfur metabolism and anti-ROS enzymes at (**A**) the 0-m depth and (**B**) the 5-m depth during the lake treatment in August. The relative abundances of the selected KEGG orthologs or pathways were predicted by PICRUSt2 (circles) or Tax4Fun2 (triangles). The PICRUSt2 predictions are shown as clr-transformed counts while the Tax4Fun2 predictions are shown as log_2_-transformed percentages. Columns show the average of the predicted relative abundances of *n* = seven biological replicates for the first time point (t = −24 h) at the 0-m depth, *n* = six biological replicates for the first time point at the 5-m depth and *n* = four biological replicates for all the other time points.

Both the Tax4Fun2 and PICRUSt2 analysis indicated that the H_2_O_2_ treatment of the lake had only a minor impact on the important ecological functions of the microbial community, such as nitrogen and sulfur cycling. Specifically, the predicted relative abundances of the functional pathways (KEGG orthologs) for the production of glycoside hydrolases, nitrogen metabolism, sulfur metabolism and anti-ROS activity all remained stable at both depths in response to the lake treatment (Figure 9).

**Figure 10:**
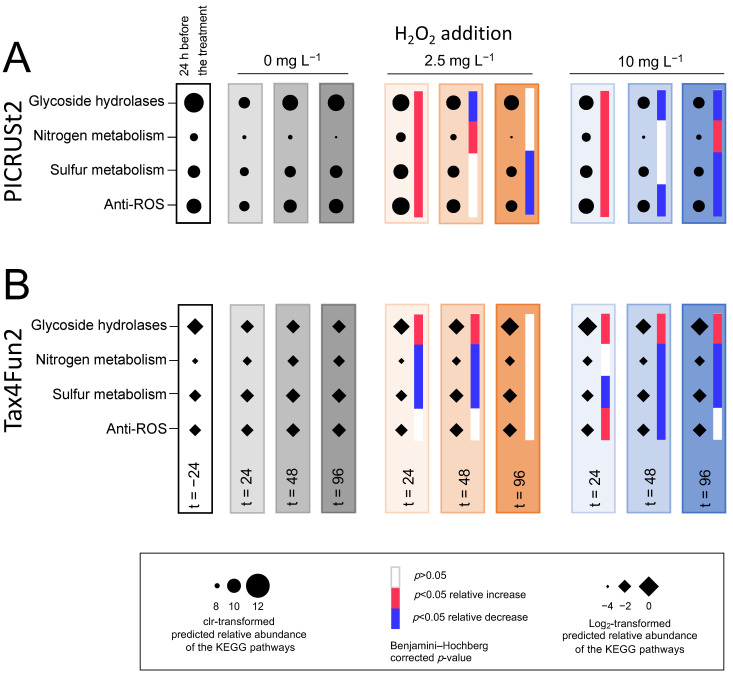
Functional prediction of the microbial community during the incubation experiments in August. The graphs show relative abundances of the selected KEGG orthologs or pathways for glycoside hydrolases, nitrogen and sulfur metabolism and anti-ROS enzymes during the incubation experiments in August as predicted by (**A**) PICRUSt2 and (**B**) Tax4Fun2. The first column presents relative abundances of the KEGG pathways in the lake prior to the incubation experiments (t = −24 h, with *n* = seven biological replicates). The other columns present relative abundances in the incubation experiments at the three subsequent time points (t = 24, 48 and 96 h, with *n* = four biological replicates per time point). Gray columns represent the control incubations (0 mg L^−1^ H_2_O_2_), orange columns—the 2.5 mg L^−1^ H_2_O_2_-treated incubations, blue columns—the 10 mg L^−1^ H_2_O_2_-treated incubations. The color intensities of the columns represent different time points. Relative abundances are shown as (**A**) clr-transformed counts for PICRUSt2 and (**B**) log_2_-transformed percentages for Tax4Fun2. Significant differences in relative abundances at the same time point between the treated and control incubations were tested using (**A**) ALDEx2 and (**B**) the Wilcoxon test (see the Materials and Section 2 for details). Significant increases compared to the control are shown in red, significant decreases—in blue, in the heat map next to each time point. White fields in the heat map indicate that the relative abundances were not significantly different between the treated and control incubations.

In the incubation experiments, several minor but significant differences were found between the H_2_O_2_ treatments and the control (Figure 10). At 24 h, glycoside hydrolases and anti-ROS orthologs showed significantly higher relative abundances in the incubations treated with H_2_O_2_ than in the control according to both PICRUSt2 and Tax4Fun. Other significant differences were less consistent among the two pipelines. For instance, the relative abundance of the nitrogen metabolism was significantly higher in the H_2_O_2_ treatments than in the control according to PICRUSt2 (Figure 10A), whereas it was significantly lower according to Tax4Fun (Figure 10B). Overall, the predicted relative abundances of the functional pathways did not show major differences between the H_2_O_2_ treatments and the control.

### 3.6. Comparison of the June and August Treatments

The lake treatments in June and August differed in subtle ways. The water temperature was lower and oxygen penetrated slightly deeper in June than in August (Figure S3). Furthermore, the measured H_2_O_2_ concentrations were slightly lower, and H_2_O_2_ degraded faster during the lake treatment in June (Figure S1) than during the lake treatment in August (Figure 2). The N and P nutrient dynamics were comparable for both lake treatments, with NH_4_^+^ concentrations strongly increasing after the addition of H_2_O_2_ in both the June and August treatments while NO_x_ and PO_4_^3−^ were much less affected (compare Figures S4 and S7). The phytoplankton community in June was largely dominated by the cyanobacterium *Aphanizomenon klebahnii*. Despite the fast H_2_O_2_ degradation, the June treatment led to an 89% decline of the biovolume of *A. klebahnii* within 48 h after H_2_O_2_ addition (Figure S8A,B).

The bacterial abundances in June were higher at 0 m than at the 5-m depth (in contrast to August) and peaked 1 day later (Figure S9A). Most of the taxa that increased in relative abundance after the lake treatment in June (Flavobacteriales, Rhodobacterales, Alteromonadales and Pseudomonadales; Figure S10) were similar to those that increased in relative abundance after the lake treatment in August (Figure 5). In particular, the genera *Flavobacterium* (Flavobacteriales) and *Rheinheimera* (Alteromonadales) again codominated the microbial community at 24 h after H_2_O_2_ addition and again fell back to lower relative abundances during the subsequent days (Figure S11). Conversely, the taxa displaying a distinct decrease in the relative abundance after the June treatment (Bdellovibrionales and Bacteroidales at the 5-m depth) differed from the taxa that decreased after the August treatment (compare Figure 5 and Figure S10).

In the incubation experiments, many of the taxa responding significantly to the H_2_O_2_ treatments in June differed from those in August (compare Figure 6 and Figure S12). However, some taxa showed a more consistent pattern. Specifically, the genera *Rheinheimera* (Alteromonadales) and *Pseudomonas* (Pseudomonadales) reached significantly higher abundances in the H_2_O_2_ treatments than in the control, whereas Verrucomicrobia of the orders Chthoniobacterales and Pedosphaerales reached significantly lower abundances in response to H_2_O_2_ in both June and August (Figures S12 and S13).

Alpha diversity indices of species richness and phylogenetic diversity were similarly affected after the June and August treatments, with an initial decline during the first few days, followed by a strong recovery (Figure S14). As indicated by the beta diversity analysis, the lake microbial community composition in June also recovered, although beta diversity trajectories of the incubation experiments showed somewhat less convergence between the H_2_O_2_ treatments and the control in June than in August (Figure S15). Similarly to August, the predicted relative abundances of the functional pathways were not strongly affected during the H_2_O_2_ treatment in June (Figures S16 and S17). Again, the significance patterns of the incubation experiments were not fully consistent among the two pipelines (Figure S17). At 48 h, however, glycoside hydrolases, nitrogen metabolism and anti-ROS orthologs showed significantly higher relative abundances in the incubations treated with H_2_O_2_ than in the control according to both PICRUSt2 and Tax4Fun.

## 4. Discussion

### 4.1. Effect of H_2_O_2_ on the Phytoplankton Community

The results of this study confirm that low concentrations of H_2_O_2_ can effectively suppress cyanobacterial blooms. In both treatments, dominant cyanobacteria decreased rapidly, as shown by the decline of *Aphanizomenon klebahnii* in the June treatment and of *Dolichospermum* sp. in the August treatment. A fast decline of cyanobacterial blooms has also been observed in two previous H_2_O_2_ treatments of lakes dominated by *Planktothrix agardhii* [43] and *Aphanizomenon flos-aquae* [25]. During the August treatment, *P. agardhii* codominated the cyanobacterial bloom, but showed more resistance to H_2_O_2_ than *Dolichospermum* sp. Remarkably, in the incubation experiments with a starting concentration of 10 mg L^−1^, *P. agardhii* did not decline. A possible explanation for the strong persistence of *P. agardhii* in this study compared to *Dolichospermum* sp. and to *P. agardhii* in a previous lake treatment [43] might be the genetic variation in H_2_O_2_ sensitivity between the different strains as previously observed in both *Planktothrix* spp. [92] and *Microcystis aeruginosa* [93].

Eukaryotic dinoflagellate *Ceratium* spp. was abundant in the lake during the August treatment and was less affected by H_2_O_2_ than the cyanobacterium *Dolichospermum* sp. This observation is in line with many previous studies, which showed that cyanobacteria tend to be more resistant than eukaryotic phytoplankton species [19,20,21,22,23,24,25,26]. In contrast to the lake observations, *Ceratium* spp. declined in all the incubation experiments, including the control. Apparently, keeping *Ceratium* spp. in enclosed incubation bags was detrimental for this organism. The enclosed space may have disrupted their natural migrating behavior and likely caused their decline even in the control without H_2_O_2_. *Ceratium* spp. are good swimmers and known to form subsurface maxima by vertical migration during high light conditions [94]. 

Simultaneous investigation of the zooplankton community showed that the H_2_O_2_ treatment of the lake negatively affected rotifers, small zooplankton known to graze on bacteria and small phytoplankton [95,96]. In contrast, cladocerans and copepods, which can also graze on bacteria but mainly feed on larger phytoplankton size classes [96,97], were not affected by the H_2_O_2_ concentrations administered to the lake (personal communication, Weenink and Visser).

### 4.2. Bacterial Response to the H_2_O_2_ Treatment

The bacterial abundances observed in this study before the H_2_O_2_ treatment (0.5–6 × 10^6^ cells mL^−1^; Figure 4 and Figure S9) are comparable to the earlier published values for freshwater lakes [98,99]. Overall, the bacterial numbers were not much affected by the H_2_O_2_ treatment, except for a temporary ca. threefold increase within 1–2 days after the treatment. The peak in bacterial abundance coincided with a temporary decline in alpha diversity, shifts in beta diversity, increasing ammonium concentrations and increases in the relative abundance of anti-ROS and glycoside hydrolase orthologs. Glycoside hydrolases are enzymes involved in the degradation of the polysaccharides derived from plant and algal biomass such as glycogen, cellulose and starch, whereas ammonium is released by the degradation of N-rich molecules such as proteins. Hence, these results indicate that some bacteria with adequate H_2_O_2_ protection mechanisms temporarily profited from the degradation of organic compounds released by the lysing cyanobacterial bloom. The subsequent return to lower bacterial abundances might be a consequence of the temporary nature of the lysis event, additional losses (grazing), altered organic carbon sources due to the strongly changed phytoplankton composition or linked to the changing environmental conditions in the lake (such as the decline in temperature, oxygen saturation and pH) [100].

### 4.3. Effect of H_2_O_2_ on Microbial Community Composition

Many of the dominant bacterial phyla of lake Oosterduinse Meer detected by our primer set are also common in other freshwater lakes around the world [22,26,101,102,103,104]. However, the microbial community composition of lakes is known to vary substantially throughout the seasons and often in accordance with changing environmental conditions [98,102,103,105], as reflected by the differences in community composition between both treatments of this study. Hence, the impacts of H_2_O_2_ treatments should be interpreted against the background of natural changes in microbial community composition.

Some taxa consistently increased in the relative abundance after exposure to H_2_O_2_, both in the lake treatments and in the controlled incubation experiments. In particular, several members of the Proteobacteria strongly increased in relative abundance, as also observed in the recent studies of Lusty and Gobler [26] and Santos et al. [42]. In our study, these members included Alteromonadales (Gammaproteobacteria) of the genus *Rheinheimera*, which have been found to produce H_2_O_2_ themselves [106], as well as Pseudomonadales, which have also been described to be very resistant against oxidative stress [107]. Both *Rheinheimera* and Pseudomonadales also increased in relative abundance after H_2_O_2_ treatments in the field mesocosm study of Lin et al. [22]. Amongst Alphaproteobacteria, Rhodobacterales repeatedly increased in relative abundance after H_2_O_2_ exposure in this study, which is in line with the results in the 2 mg L^−1^ H_2_O_2_-treated field mesocosms of Lin et al. [22] and the increase of *Paracoccus* (Rhodobacteraceae) in the mesocosm experiments of Santos et al. [42]. This is not surprising given that members of Rhodobacterales master a wide range of growth mechanisms, from photoautotrophy and (an-)aerobic respiration to fermentation, and this flexibility may enable them to rapidly respond to changing environmental conditions [108,109,110]. Contrary to our results, both Lusty and Gobler [26] and Santos et al. [42] reported a decrease in the relative abundance of Actinobacteria in outdoor incubation experiments with lake water after addition of H_2_O_2_. While small decreases in relative abundance were observed for Microtrichales during the June incubations and after the August lake treatment, most orders of the phylum Actinobacteria were rather stable or increased in response to the H_2_O_2_ treatments (e.g., the order of Frankiales).

Only a few taxa showed a consistently lower relative abundance after exposure to H_2_O_2_ in the incubation experiments, including three orders of the phylum Verrucomicrobia (i.e., Chthoniobacterales, Pedosphaerales and, to a lesser extent, Verrucomicrobiales). This agrees with previous observations that Verrucomicrobia suffered from H_2_O_2_ treatments [22,26,42]. However, the increase in the relative abundance of Chthoniobacterales within the lake two to four days after the August treatment (Figure 5) seems to contrast their decline in the incubation experiments (Figure 6). Chthoniobacterales belong to Spartobacteria, a class of heterotrophic Verrucomicrobia inhabiting soils and aquatic environments [111,112]. A metagenomic study in the Baltic Sea found that Spartobacteria produced a large diversity of glycoside hydrolases targeting a variety of carbohydrates and observed a close association of Spartobacteria with cyanobacteria that may have produced carbohydrate substrates [113]. Hence, a potential explanation for our observations might be that Chthoniobacterales are first temporarily suppressed by H_2_O_2_, but benefit from storage and structural polysaccharides [114] released by the lysing cyanobacterial bloom once the added H_2_O_2_ has disappeared.

### 4.4. Community Resilience after the Treatment with H_2_O_2_

Changes in the microbial community in response to disturbances are best described by the terms “resistance” and “resilience.” Resistance describes the degree to which a community can remain unchanged during a disturbance, whereas the resilience of a community describes the rate of recovery after a disturbance [115]. Disturbances to the ecosystem can have both positive and negative effects on alpha diversity [102,116,117] and sometimes even opposite effects in the epilimnion and hypolimnion of the same lake [100]. The microbial communities in the lake and the incubation experiments were not resistant to the H_2_O_2_ treatments as indicated by a strong decrease in species richness as well as phylogenetic diversity (Figure 7 and Figure S14), but alpha diversity also recovered within a few days. This temporary response was also visible in the shifts in community composition in both the lake treatment and the incubation experiments, where *Rheinheimera* and *Flavobacterium* strongly increased in relative abundance during the first 24 h after H_2_O_2_ addition but subsequently declined again. The temporary negative effect on alpha diversity and concomitant shift in microbial community composition might be explained by the very nature of H_2_O_2_ treatments. Unlike most other chemical treatments, added H_2_O_2_ rapidly degrades to water and oxygen and hence disappears from the lake within a few days. In ecological terms, H_2_O_2_ treatments thus represent pulse (short-term) disturbances rather than press (long-term) disturbances [118,119]. After the added H_2_O_2_ has disappeared, the microbial community can recover. Strong resilience of the species richness and phylogenetic diversity observed in the lake and in the incubations indicates that most taxa survived the applied concentrations of H_2_O_2_ and were temporarily suppressed rather than removed from the ecosystem. Similar alpha diversity resilience patterns of microbial communities have been reported for a variety of other ecosystems, including freshwater lakes, after other pulse disturbances [100,115,120].

High resilience after the H_2_O_2_ treatments was also visible from the analysis of beta diversity in the lake (Figure 8 and Figure S11). The microbial communities recovered mostly in a V-shaped pattern for both the dissimilarity and the distance matrices. This indicates that the community after the lake H_2_O_2_ treatment did not return to the exact same starting point but rather to a slightly altered community composition similar to the previously observed after other pulse disturbances [100,121]. Most likely, this V-shaped recovery is caused by altered environmental conditions during the days after the treatment such as a change in weather conditions leading to an increased mixing depth of the lake [100,104]. In contrast to laboratory or incubation experiments, spatial refuges for bacteria in lakes likely facilitate the resilience of microbial communities [121], e.g., close to the sediment in shallow parts of the lake where H_2_O_2_ degradation rates are strongly increased.

In contrast to whole lake treatment, incubation treatments allow for experimental replication and the inclusion of controls without H_2_O_2_. The beta diversity analysis of the incubation experiments confirmed the V-shaped recovery pattern observed in the lake. In addition, it showed that not only the treated communities were shifting towards a new, altered composition, but also the community composition of the untreated control communities was shifting in the same direction. While it remains uncertain to what extent the shift of the control communities was due to natural processes or, rather, due to the so-called “bottle effect” [122], the convergence of the trajectories of the treated and control communities demonstrates strong resilience of the communities after H_2_O_2_ additions.

### 4.5. Microbial Functions Show Resistance after the Treatment with H_2_O_2_

H_2_O_2_ had only minor effects on the functional pathways investigated in this study. The strong resistance to H_2_O_2_ treatments indicated by the prediction of stable relative abundances of the functional pathways (KEGG orthologs) by both pipelines is likely due to redundancy in functionality within microbial communities [115,123].

It is important to point out that the functional predictions were not always consistent between both pipelines and therefore require careful interpretation. Functional predictions based on the 16S rRNA gene amplicon sequencing data have strongly improved in recent years, including due to expanded reference databases of pipelines like Tax4fun2 and PICRUSt2 [86,87]. However, the prediction accuracy is tightly linked to the coverage of those reference databases, in which freshwater environments are still strongly underrepresented [86,124]. For more accurate analyses of the functional profiles of freshwater microbial communities, metagenomic and metatranscriptomic studies would be required. Despite the increasing use of metagenome reference databases to assess microbial functions in freshwater environments [125,126,127,128], a strong degree of uncertainty of the functional predictions is so far unavoidable. 

Both pipelines indicated that the H_2_O_2_ treatments were accompanied by a temporary increase in the relative abundance of the pathways representing anti-ROS enzymes and glycoside hydrolases. The consistency of this pattern, which was apparent in the incubation experiments in both June and August, suggests that this result is robust. A temporary increase of taxa with the anti-ROS pathways signifies that effective H_2_O_2_ defense mechanisms play a role in the resistance to H_2_O_2_ treatment [129]. In addition, possessing glycoside hydrolases to break down organic matter released by the decaying cyanobacterial bloom undoubtedly offers a clear advantage during the first few days after the treatment [56,114,130].

## 5. Conclusions

The whole lake H_2_O_2_ treatments effectively suppressed the dominant cyanobacteria *Aphanizomenon klebahnii*, *Dolichospermum* sp. and, to a lesser extent, *Planktothrix agardhii*. Analysis of the microbial community composition with primers that do not target cyanobacteria revealed that the H_2_O_2_ concentrations used in this study had a distinct temporary effect on the microbial community. The taxa that are known to be resistant to oxidative stress (e.g., *Rheinheimera*) were favored during the first 24 h after H_2_O_2_ addition, but subsequently their relative abundance declined again. The applied H_2_O_2_ concentrations thus represented a short-term pulse disturbance, which caused a temporary decline in alpha and beta diversity and a temporary increase of the functional pathways encoding anti-ROS defenses and glycoside hydrolases. However, the microbial community proved to be resilient and recovered a few days after the treatments.

## Data Availability

The sequence data for this study were deposited in the European Nucleotide Archive (ENA) at the EMBL-EBI under accession number PRJEB44985 (https://www.ebi.ac.uk/ena/browser/view/PRJEB44985; accessed on 6 April 2021).

## Supplementary Materials

The following are available online at https://www.mdpi.com/article/10.3390/microorganisms9071495/s1, Figure S1: H2O2 treatment of the lake, Figure S2: Run-to-run variation, Figure S3: Environmental variables measured during the H2O2 treatments, Figure S4: Dissolved inorganic nutrients during the H2O2 treatment in August, Figure S5: Microbial community composition at the genus level during the lake treatment in August, Figure S6: Microbial community composition at the genus level during the incubation experiments in August, Figure S7: Dissolved inorganic nutrients during the H2O2 treatment in June, Figure S8: Phytoplankton composition during the H2O2 treatment in June, Figure S9: Total bacterial abundances during the H2O2 treatment in June, Figure S10: Microbial community composition at the order level during the lake treatment in June, Figure S11: Microbial community composition at the genus level during the lake treatment in June, Figure S12: Microbial community composition at the order level during the incubation experiments in June, Figure S13: Microbial community composition at the genus level during the incubation experiments in June, Figure S14: Alpha diversity of the microbial community during the H2O2 treatment in June, Figure S15: Beta diversity of the microbial community during the H2O2 treatment in June, Figure S16: Functional prediction of the microbial community during the lake treatment in June, Figure S17: Functional prediction of the microbial community during the incubation experiments in June, Table S1: Overview of the accession numbers and metadata of the 16S rRNA amplicon sequences deposited at the ENA, Table S2: Weather data during the treatments in June and August, Table S3: Feature tables of the microbial community at the genus level for the lake treatments and the incubation experiments.
