# Supplementary material for: Resilience of Microbial Communities after Hydrogen Peroxide Treatment of a Eutrophic Lake to Suppress Harmful Cyanobacterial Blooms
Source: Microorganisms. 2021 Jul 13;9(7):1495. doi: 10.3390/microorganisms9071495 (PMC8304526; doi:10.3390/microorganisms9071495)
Supplement: Supplementary file 1 [file microorganisms-09-01495-s001.zip › Supplementary materials_microorganisms-1249397_v8.3_12-07-2021.pdf]

Supplementary materials

Resilience of microbial communities after hydrogen peroxide treatment of a eutrophic lake to suppress harmful cyanobacterial blooms

Tim Piel [1,‡], Giovanni Sandrini [1,‡], Gerard Muyzer [1], Corina P.D. Brussaard [1,2], Pieter C. Slot [1], Maria J. van Herk [1], Jef Huisman[1], Petra M. Visser [1,*]

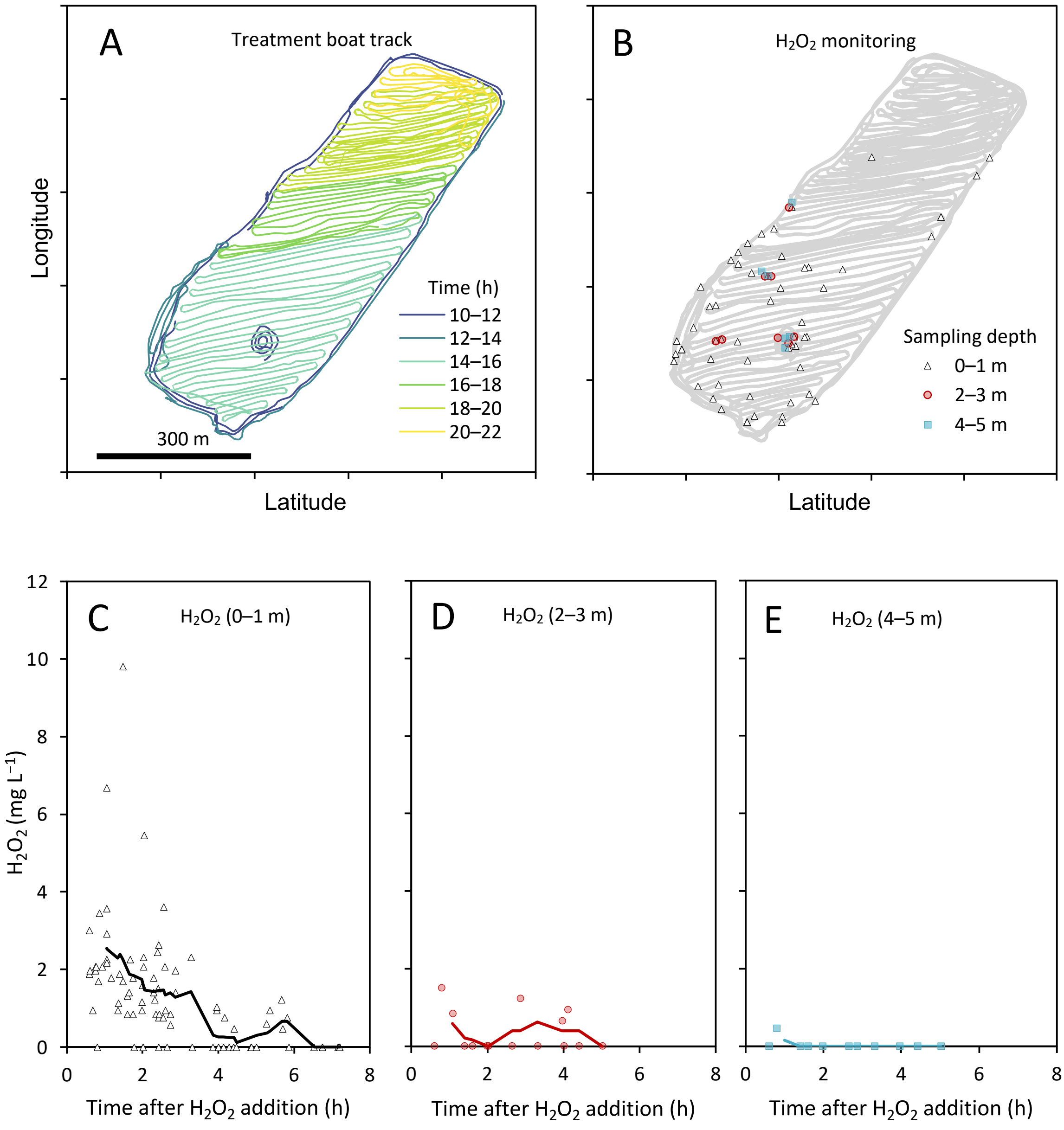

Figure S1. H_2O_2 treatment of the lake. The graph shows (**A**) the boat track during the lake treatment in June, (**B**) the sampling locations and sampling depth where H_2O_2 concentrations were monitored during the treatment. (**C-E**) H_2O_2 concentrations measured at different time points after the treatment boat had passed the sampling locations, (**C**) at 0-1 m depth, (**D**) at 2-3 m depth and (**E**) at 4-5 m depth. Colors in panel **A** indicate the time of day during which a certain section of the lake was treated, different symbols in panel **B** indicate the sampling depth, lines in panels **C-E** are moving averages with a window size of 60 min.

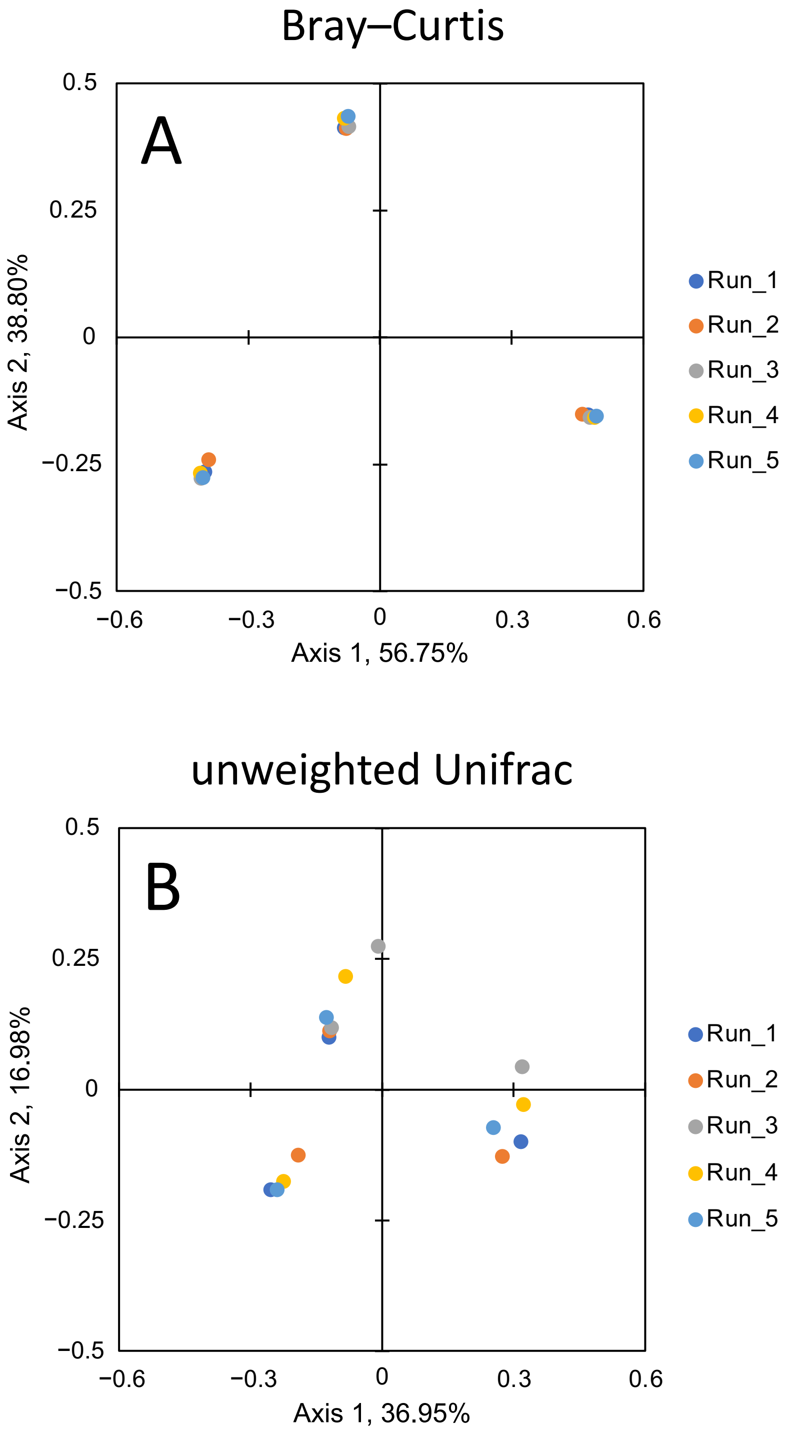

Figure S2. Run-to-run variation. PCoA plots of the beta diversity of the microbial community for three samples that were sequenced in each of the five sequencing runs. Beta diversity in (**A**) is based on the Bray-Curtis dissimilarity matrix, and in (**B**) on the unweighted UniFrac distance matrix. Each symbol in the graph represents one sequencing result, each cluster represents one of the three samples, and the five colors per cluster represent the five individual sequencing runs for that sample.

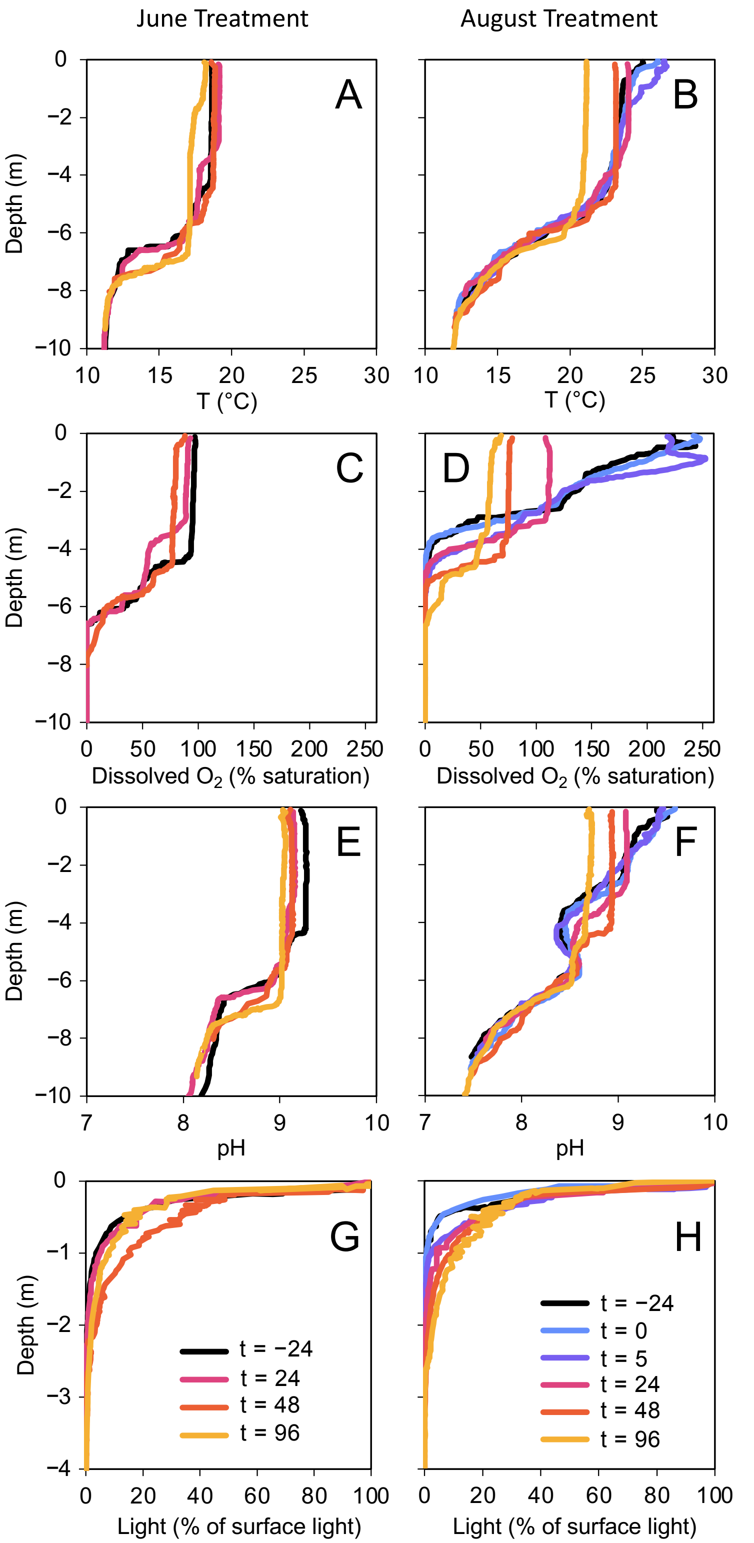

Figure S3. Environmental variables measured during the H_2O_2 treatments. The graphs show depth profiles of (**A**,**B**) temperature, (**C**,**D**) dissolved oxygen saturation, (**E**,**F**) pH and (**G**,**H**) light intensity (photosynthetically active radiation) relative to the surface during the lake treatments in June (left column) and August (right column). Colored lines indicate measurements at different time points during the treatments.

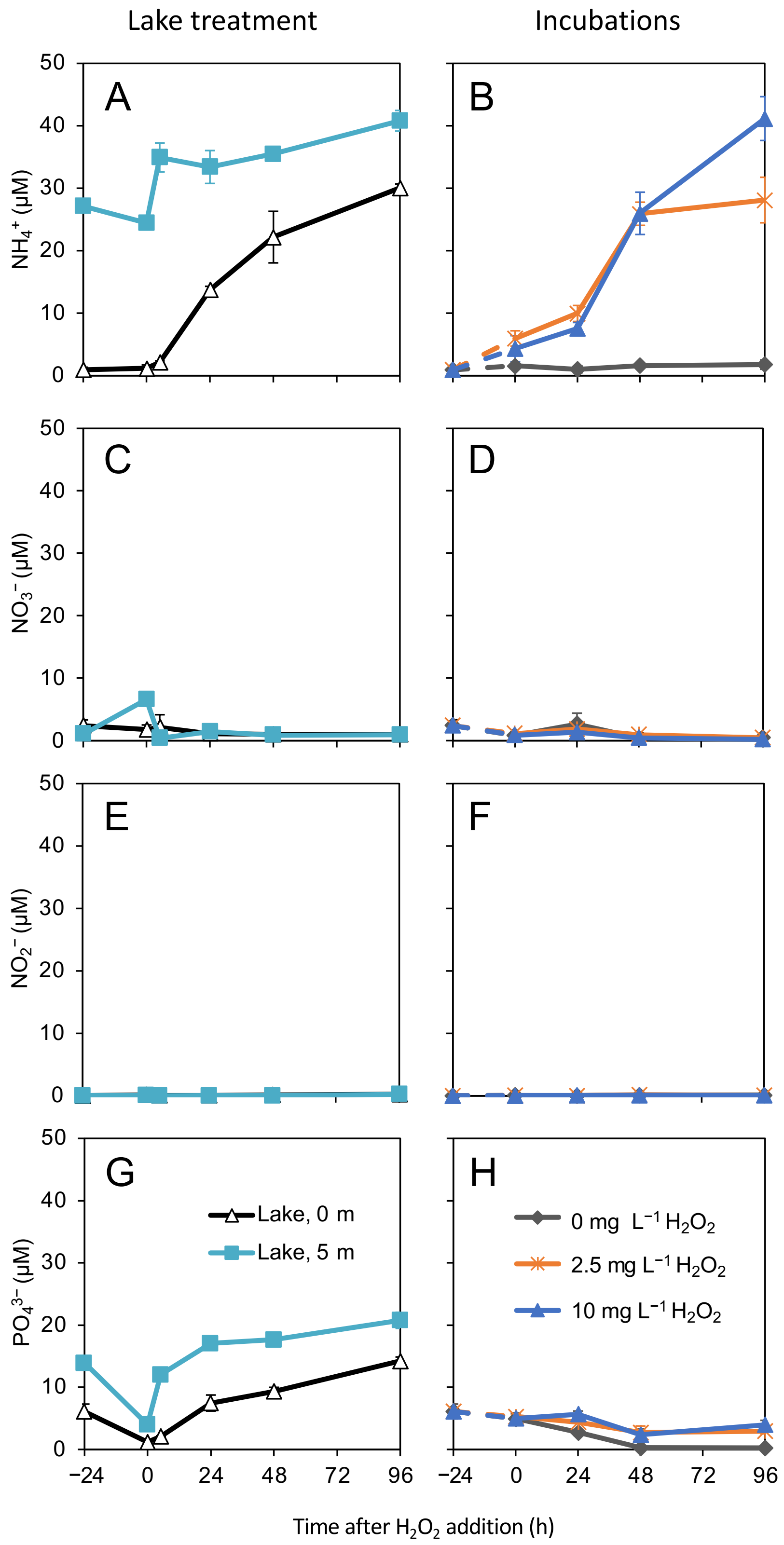

Figure S4. Dissolved inorganic nutrients during the H_2O_2 treatment in August. Concentrations of (**A,B**) NH_4^+ (**C,D**) NO_3^-, (**E,F**) NO_2^-, and (**G,H**) PO_4^{3-} measured during the lake treatment (left column) and in the incubation experiments (right column) in August. The data show the mean (± SD) of 4 biological replicates per time point.

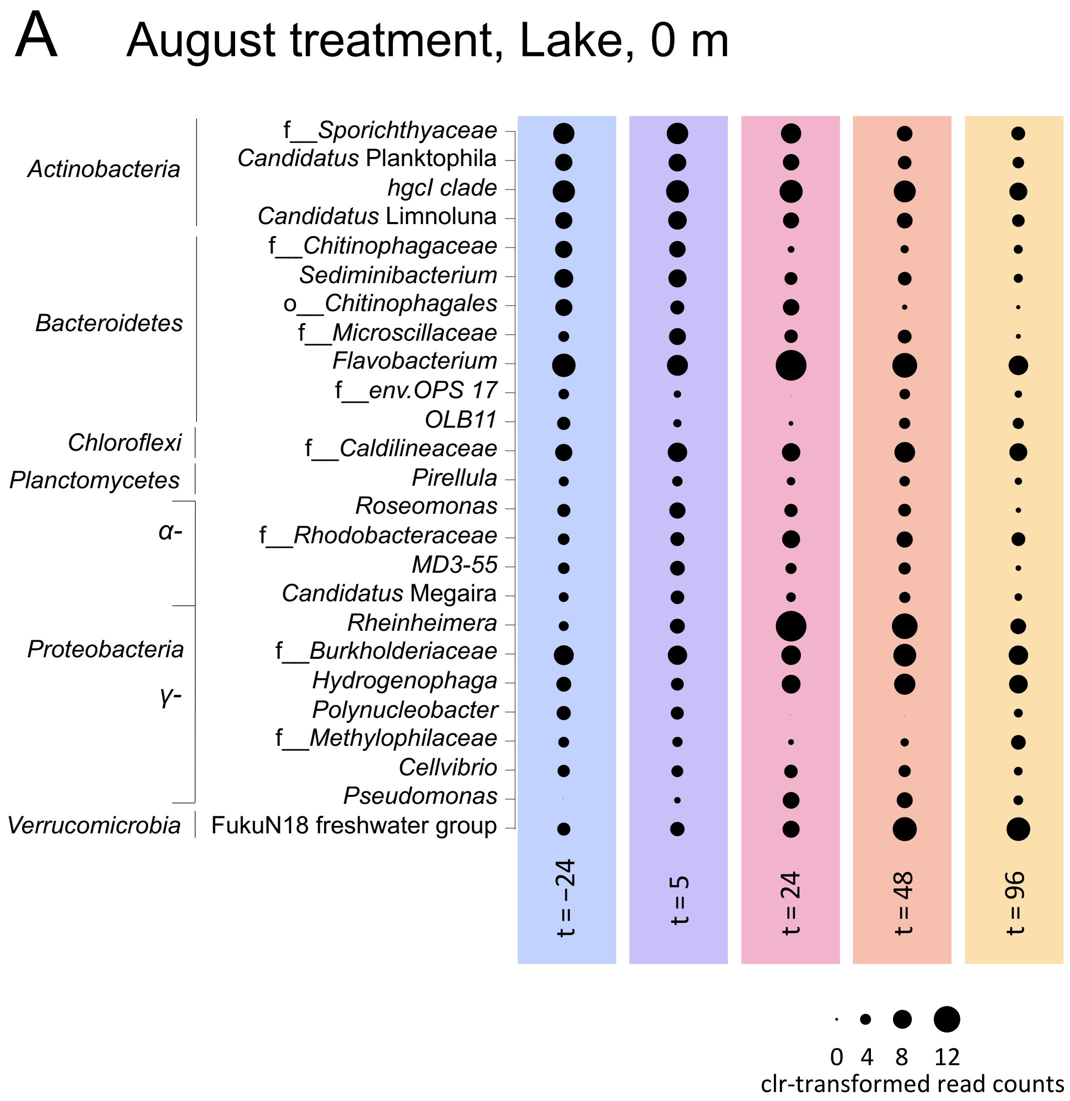

Figure S5. Microbial community composition at genus level during the lake treatment in August. Relative abundance shown as clr-transformed read counts at phylum and order level at (**A**) 0 m and (**B**) 5 m depth during the lake treatment in June. Columns represent averages of the biological replicates at each time point; columns of different colors represent different time points. "o__" and "f__" indicates that the maximum classification of these taxa is at order and family level, respectively.

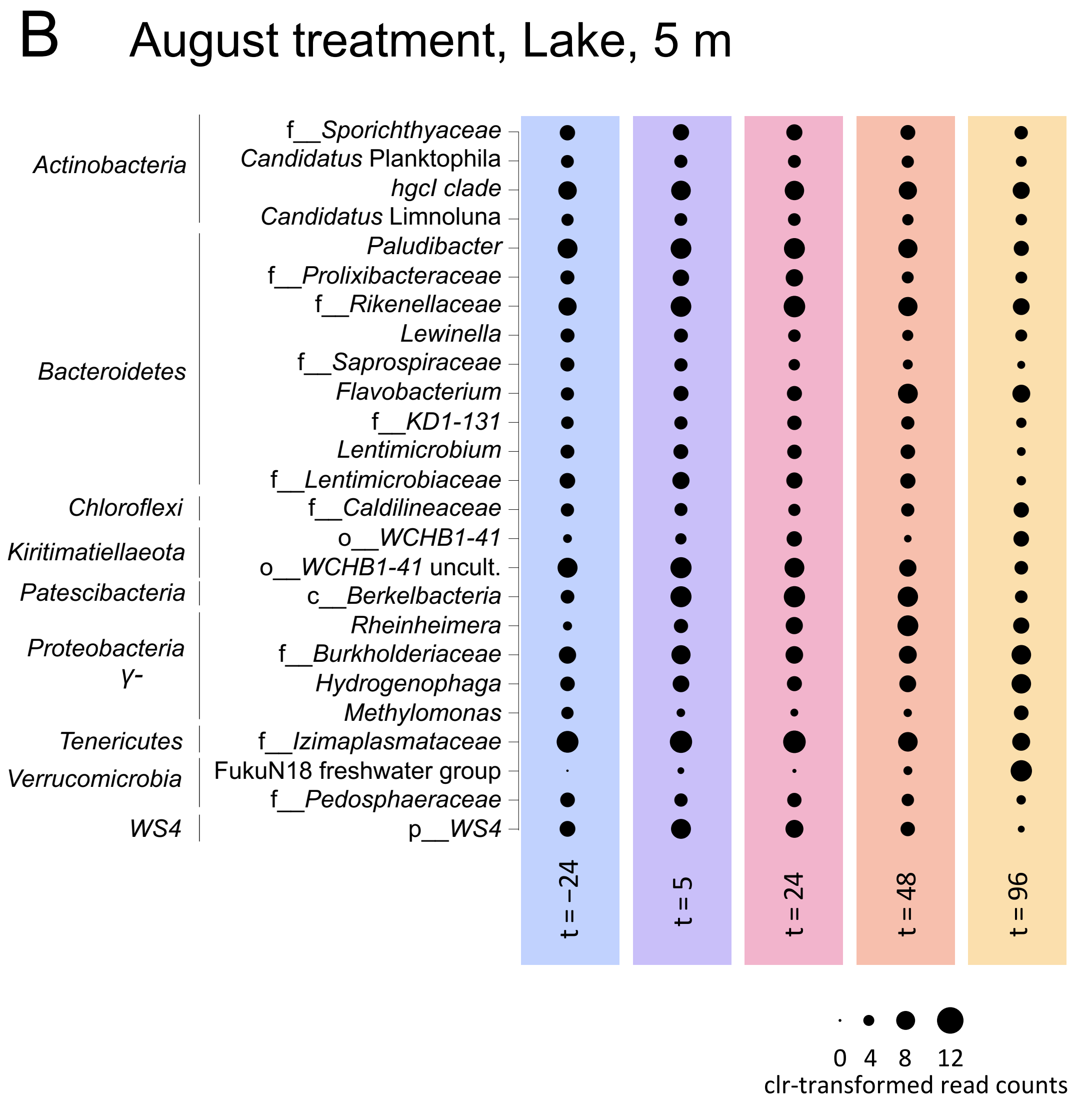

Figure S5. Continued

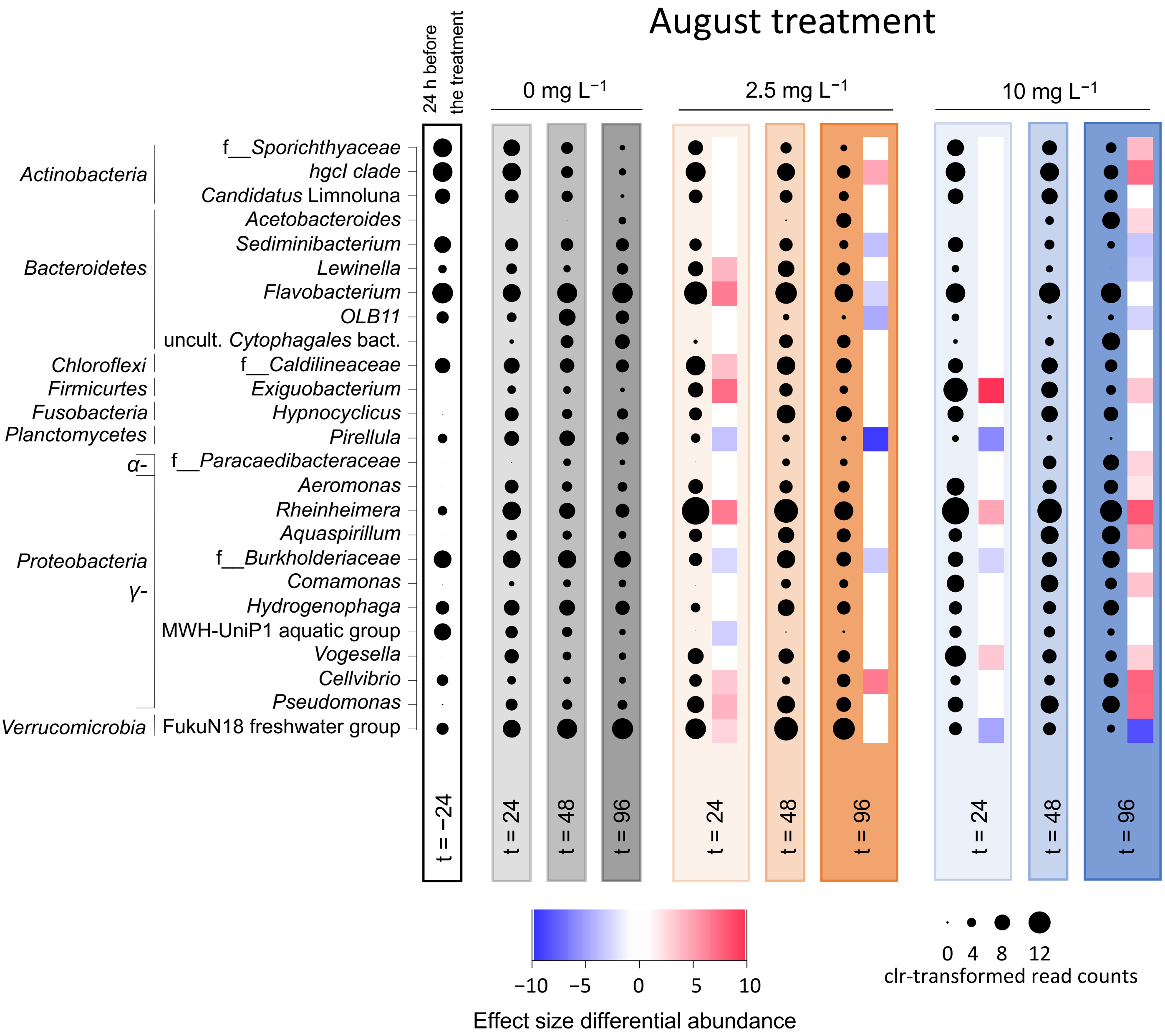

Figure S6. Microbial community composition at genus level during the incubation experiments in August. The graph shows relative abundances as clr-transformed read counts of bacteria at genus level for each of the time points during the August treatment. The first column represents the average relative abundances in the lake prior to the incubation experiments (t = -24 h, with n = 7 biological replicates). The other columns represent relative abundances in the incubation experiments at three subsequent time points (t = 24, 48 and 96 h, with n = 4 biological replicates per time point). Gray columns represent the control incubations (0 mg L^{-1} H_2O_2), orange columns the 2.5 mg L^{-1} H_2O_2 treated incubations, and blue columns the 10 mg L^{-1} H_2O_2 treated incubations. Differential abundances of orders between treated and control incubations at t = 24 h and t = 96 h were calculated using ALDEx2. Effect sizes of the statistical test (0.7 × Cohen's D) are shown in the heat map next to the t =24 and t = 96 columns for all significant comparisons; blue indicates a significant decrease in relative abundance while red indicates a significant increase in relative abundance, in comparison to the control. "f__" indicates that the maximum classification of these taxa is at family level.

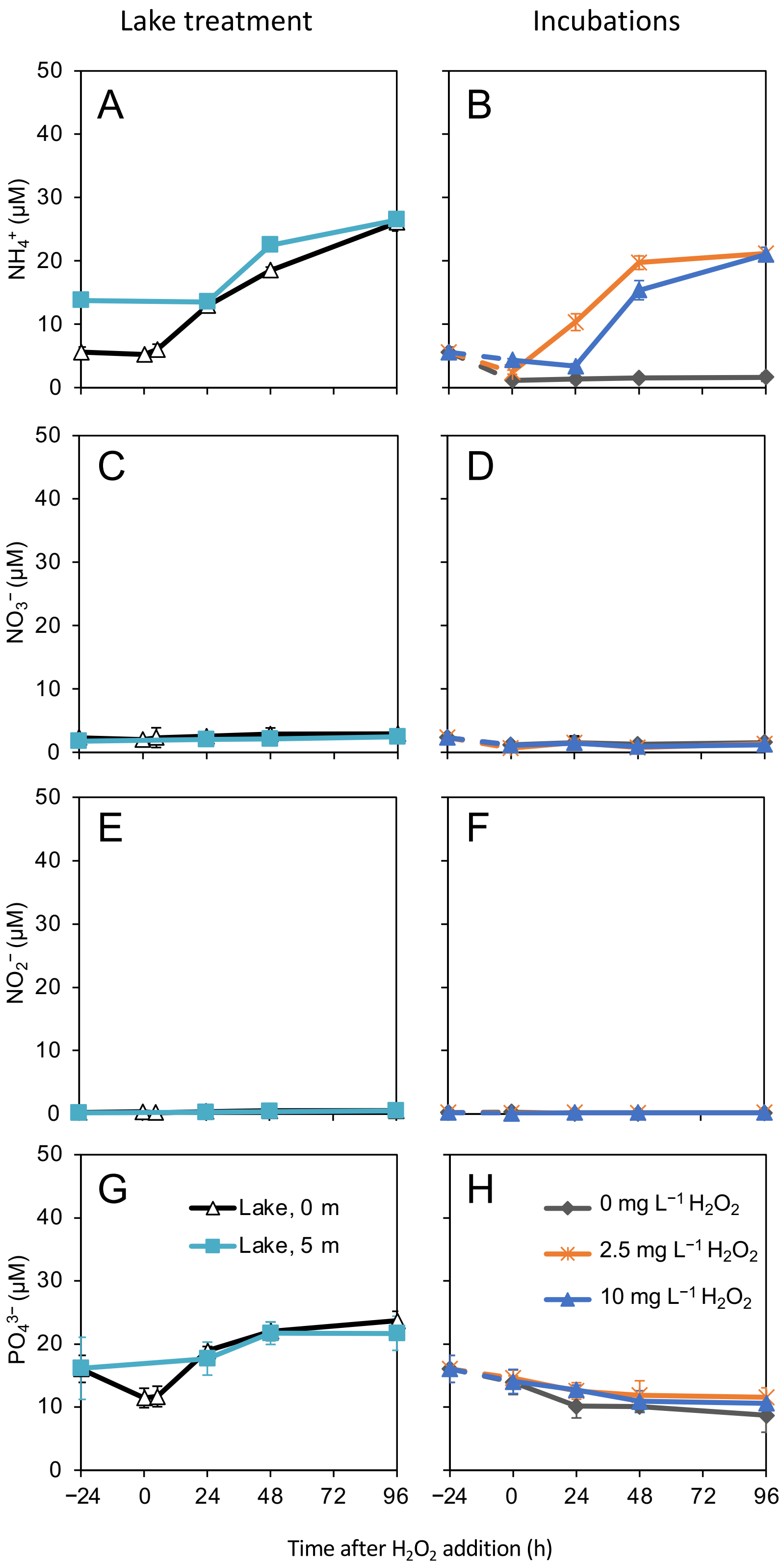

Figure S7. Dissolved inorganic nutrients during the H_2O_2 treatment in June. Concentrations of (**A,B**) NH_4^+ (**C,D**) NO_3^-, (**E,F**) NO_2^-, and (**G,H**) PO_4^{3-} measured during the lake treatment (left column) and in the incubation experiments (right column) in June. The data show the mean (± SD) of 4 biological replicates per time point.

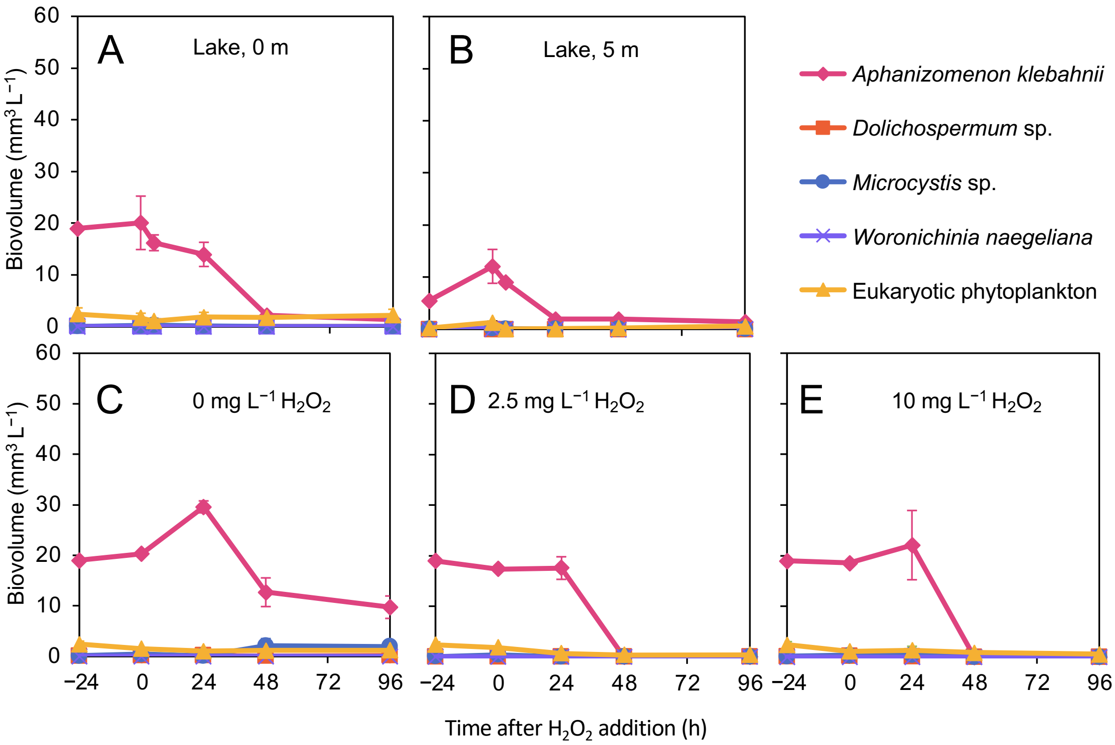

Figure S8. Phytoplankton composition during the H_2O_2 treatment in June. (**A,B**) Biovolume of the most abundant cyanobacterial taxa and of the total eukaryotic phytoplankton, at (**A**) 0 m and (**B**) 5 m depth during H_2O_2 treatment of the lake in June. (**C-E**) Biovolumes in the incubation experiments treated with (**C**) 0 mg L^{-1}, (**D**) 2.5 mg L^{-1} and (**E**) 10 mg L^{-1} of H_2O_2. The data show the mean (± SD)of 3 biological replicates per time point.

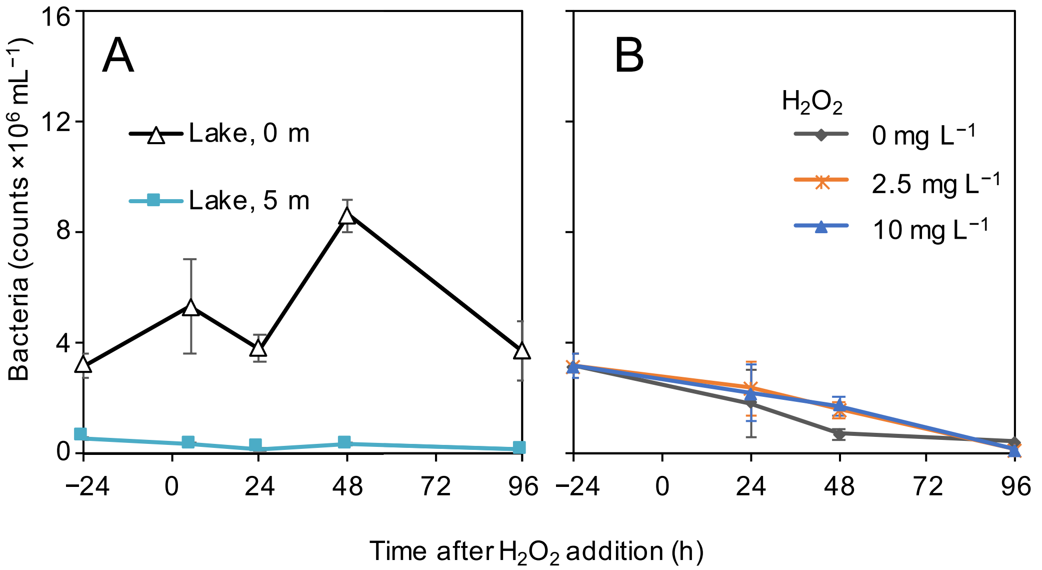

Figure S9. Total bacterial abundances during the H_2O_2 treatment in June. Total bacterial abundances as determined with flow cytometry during (**A**) the lake treatment and (**B**) incubation experiments in June. The data show the mean (± SD), based on n=7 biological replicates for t = -24 h at 0 m (**A**), n=6 biological replicates for t = -24 h at 5 m (**B**), and n=4 biological replicates for all other time points.

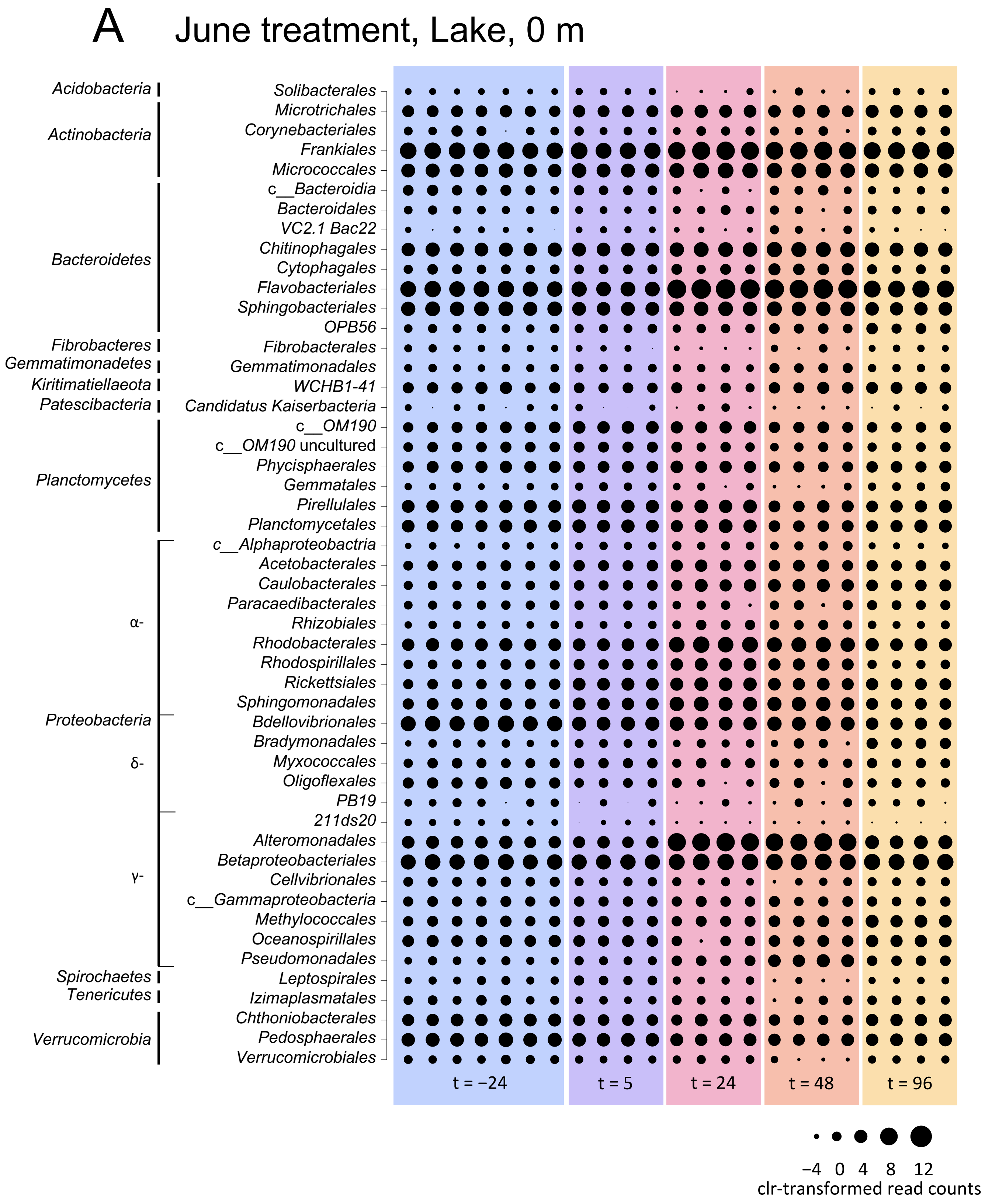

Figure S10. Microbial community composition at order level during the lake treatment in June. Relative abundance shown as clr-transformed read counts at phylum and order level at (**A**) 0 m and (**B**) 5 m depth during the lake treatment in June. Columns of the same color represent biological replicates at the same time point; columns of different colors represent different time points. "c__" indicates that the maximum classification of these taxa is at class level.

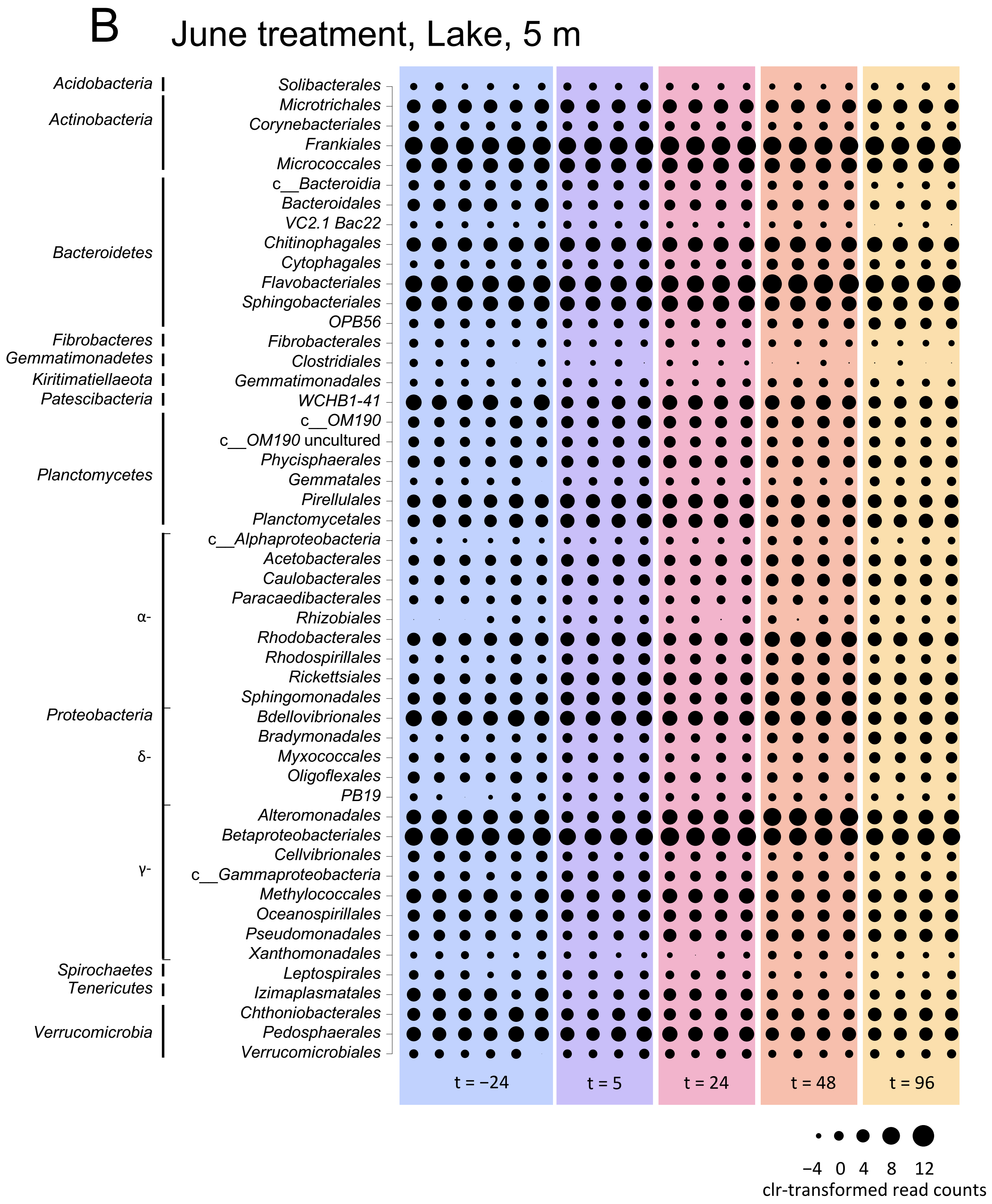

Figure S10. Continued

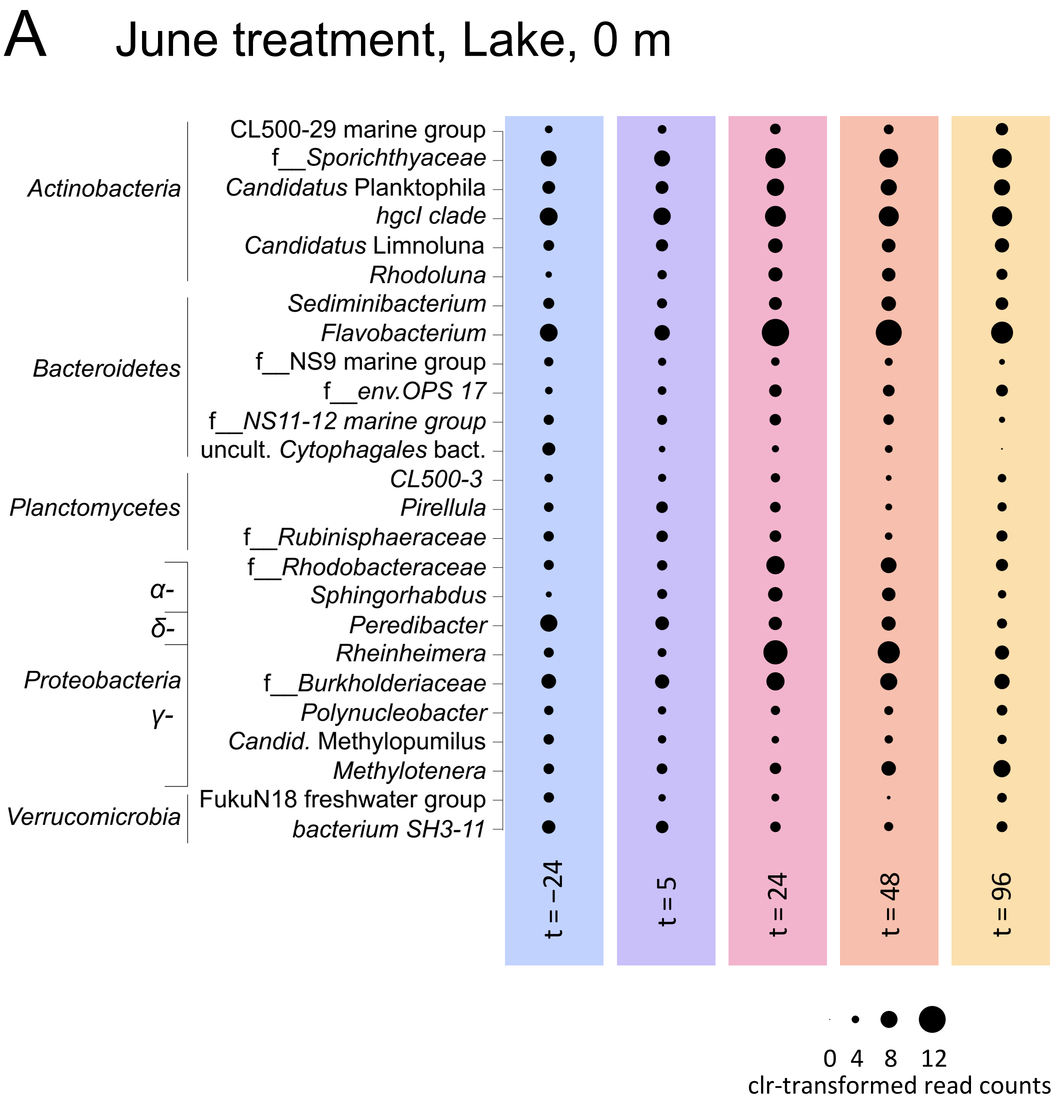

Figure S11. Microbial community composition at genus level during the lake treatment in June. Relative abundance shown as clr-transformed read counts at phylum and order level at (**A**) 0 m and (**B**) 5 m depth during the lake treatment in June. Columns represent averages of the biological replicates at each time point; columns of different colors represent different time points. "o__" and "f__" indicate that the maximum classification of these taxa is at order and family level, respectively.

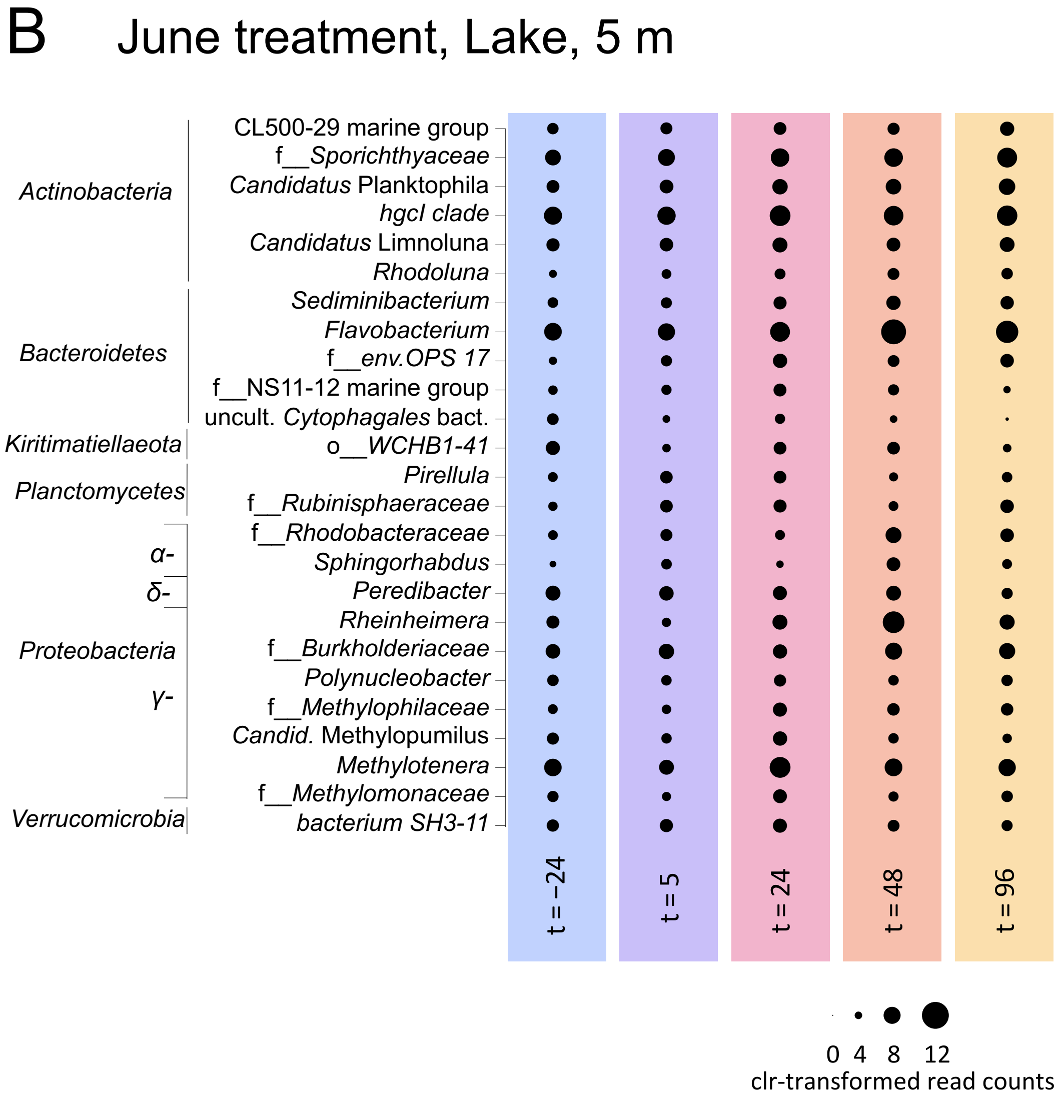

Figure S11. continued.

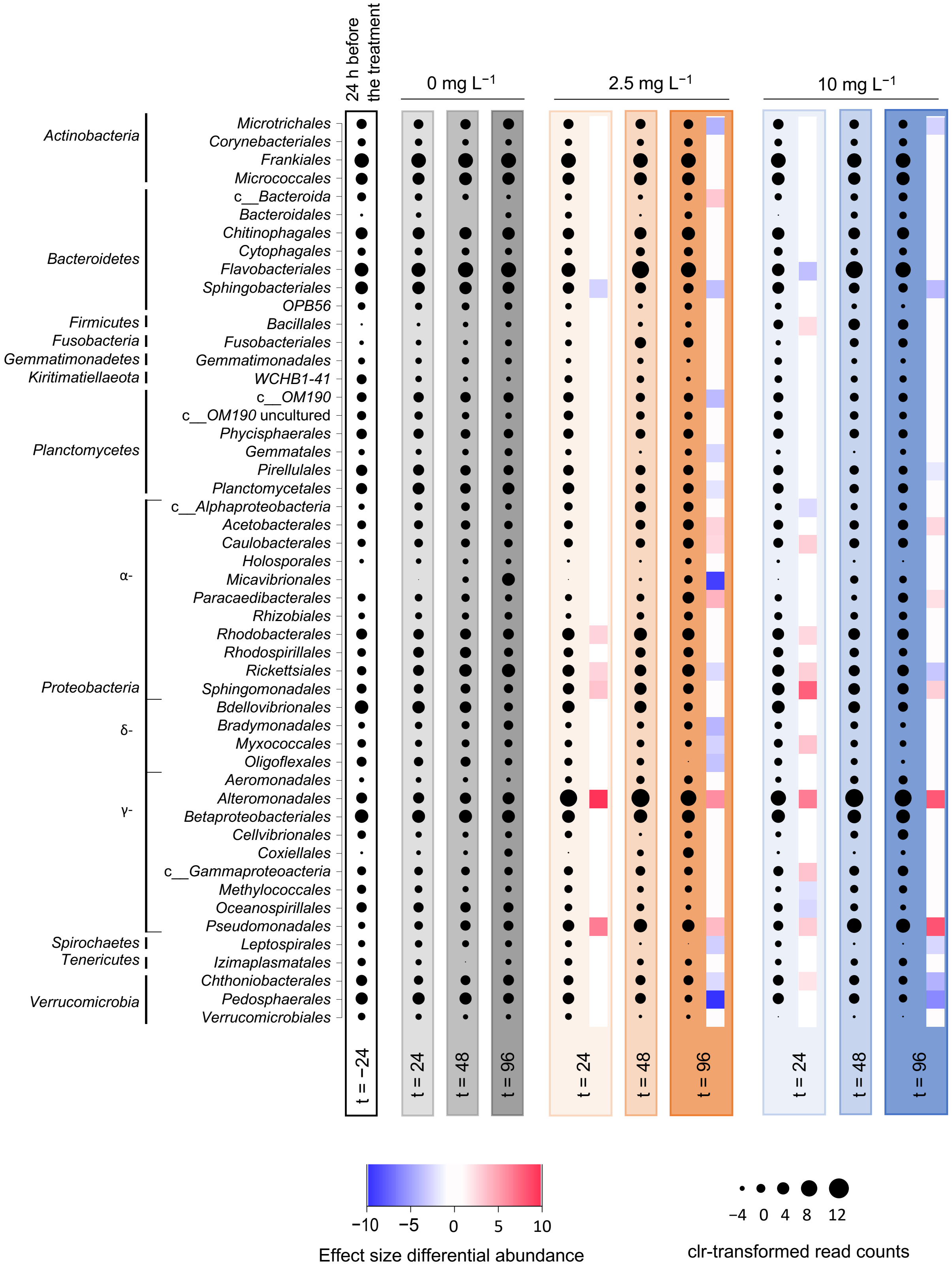

Figure S12. Microbial community composition at order level during the incubation experiments in June. The graph shows relative abundances as clr-transformed read counts of bacteria at order level for each of the time points during the June treatment. The first column represents the average relative abundances in the lake prior to the incubation experiments (t = -24 h, with n = 7 biological replicates). The other columns represent relative abundances in the incubation experiments at three subsequent time points (t = 24, 48 and 96 h, with n = 4 biological replicates per time point). Gray columns represent the control incubations (0 mg L^{-1} H_2O_2), orange columns the 2.5 mg L^{-1} H_2O_2 treated incubations, and blue columns the 10 mg L^{-1} H_2O_2 treated incubations. Differential abundances of orders between treated and control incubations at t = 24 h and t = 96 h were calculated using ALDEx2. Effect sizes of the statistical test (0.7 × Cohen's D) are shown in the heat map next to the t =24 and t = 96 columns for all significant comparisons; blue indicates a significant decrease in relative abundance while red indicates a significant increase in relative abundance, in comparison to the control. "c__" indicates that the maximum classification of these taxa is at class level.

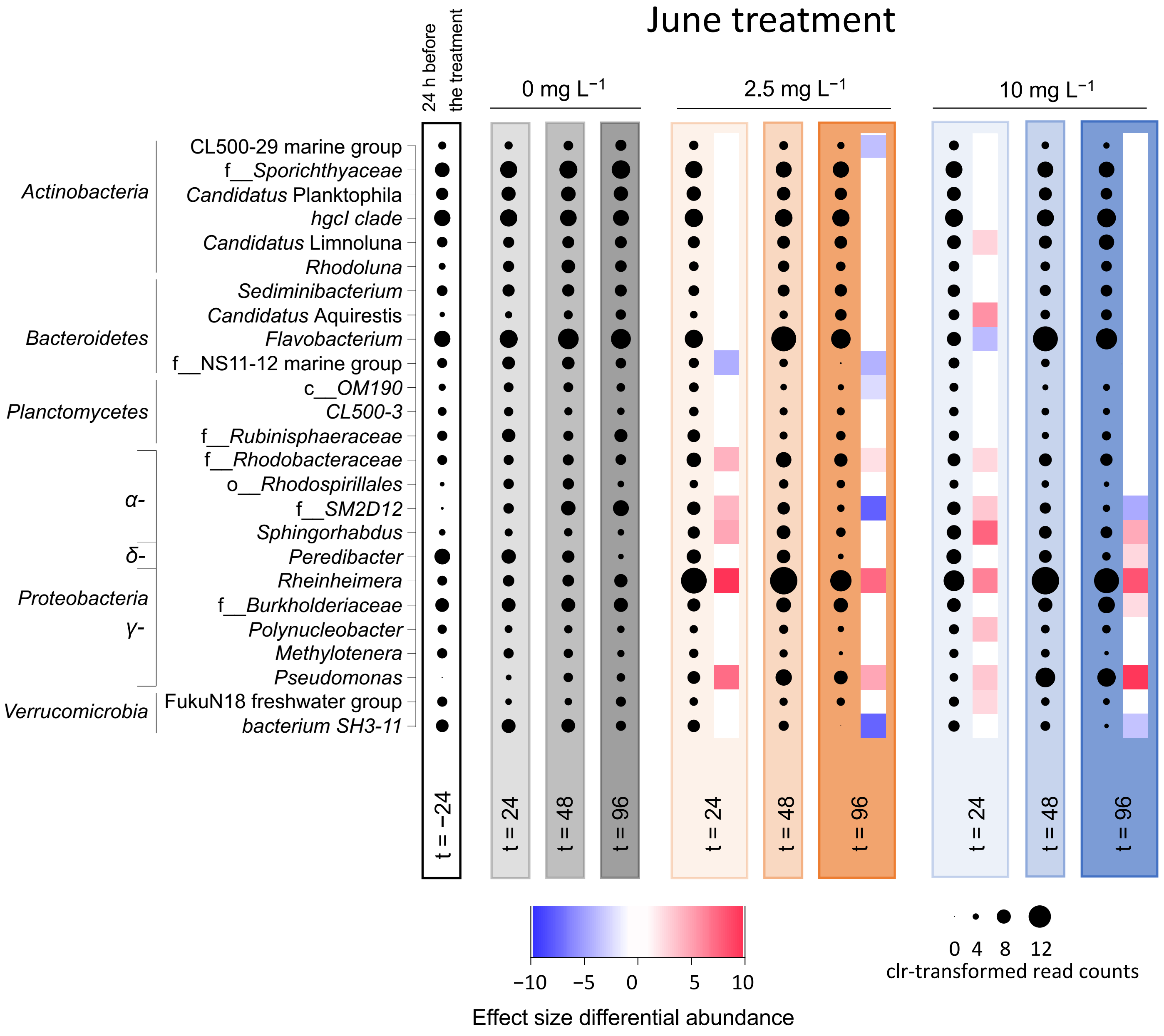

Figure S13. Microbial community composition at genus level during the incubation experiments in June. The graph shows relative abundances as clr-transformed read counts of bacteria at genus level for each of the time points during the June treatment. The first column represents the average relative abundances in the lake prior to the incubation experiments (t = -24 h, with n = 7 biological replicates). The other columns represent relative abundances in the incubation experiments at three subsequent time points (t = 24, 48 and 96 h, with n = 4 biological replicates per time point). Gray columns represent the control incubations (0 mg L^{-1} H_2O_2), orange columns the 2.5 mg L^{-1} H_2O_2 treated incubations, and blue columns the 10 mg L^{-1} H_2O_2 treated incubations. Differential abundances of orders between treated and control incubations at t = 24 h and t = 96 h were calculated using ALDEx2. Effect sizes of the statistical test (0.7 × Cohen's D) are shown in the heat map next to the t =24 and t = 96 columns for all significant comparisons; blue indicates a significant decrease in relative abundance while red indicates a significant increase in relative abundance, in comparison to the control. "c__", "o__", and "f__" indicate that the maximum classification of these taxa is at class, order, and family level, respectively.

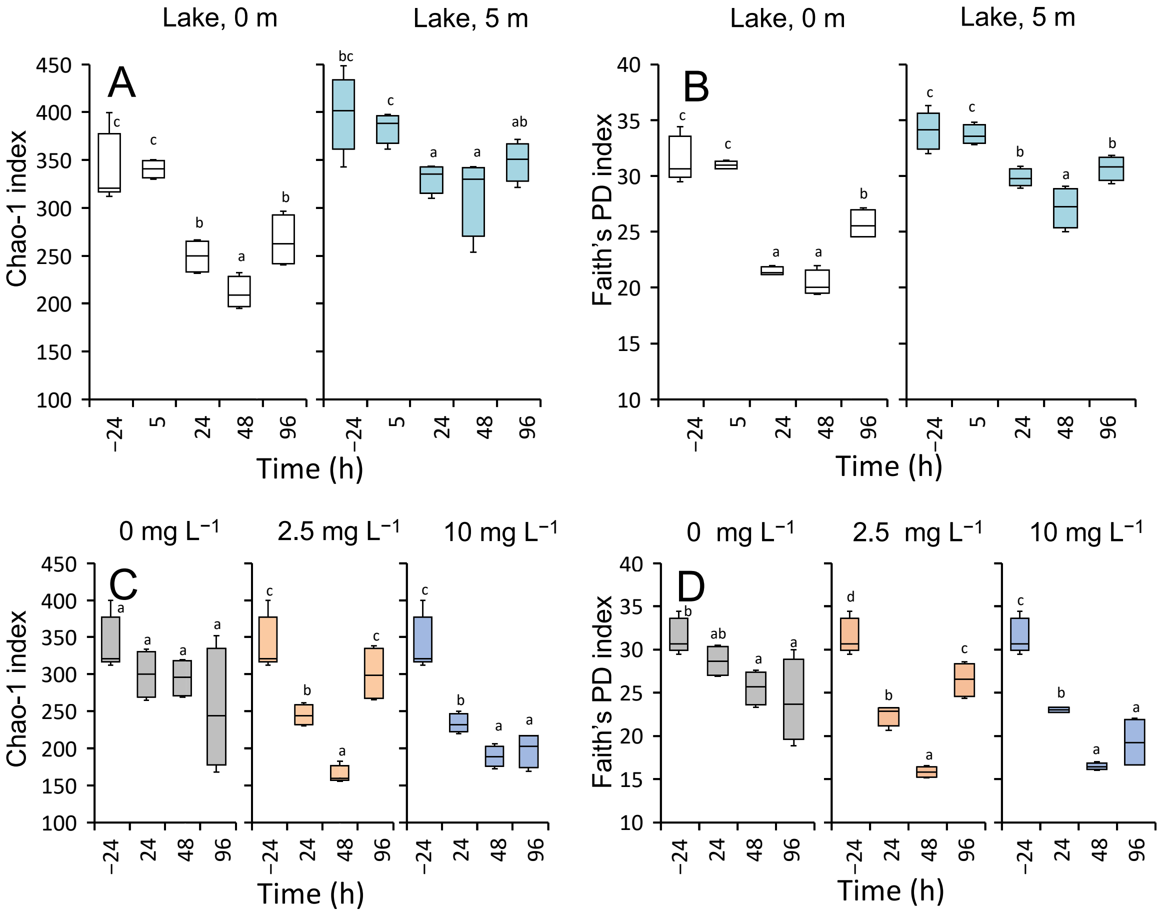

Figure S14. Alpha diversity of the microbial community during the H_2O_2 treatment in June. (**A,C**) The Chao-1 index (estimated species richness), and (**B,D**) Faith's phylogenetic diversity during (**A,B**) the lake treatment and (**C,D**) the incubation ex-periments in August. The color of the box plots indicate (**A,B**) the sampling depth in the lake or (**C,D**) the different H_2O_2 treatment concentrations of the incubations. The first box plot in each panel represents the alpha diversity in the lake prior to the incubation experiments (t = -24 h, with n = 7 biological replicates). Box plots of all subsequent time points are based on n=4 biological replicates. Box plots with different letters indicate significant differences between time points according to pairwise Kruskal-Wallis tests .

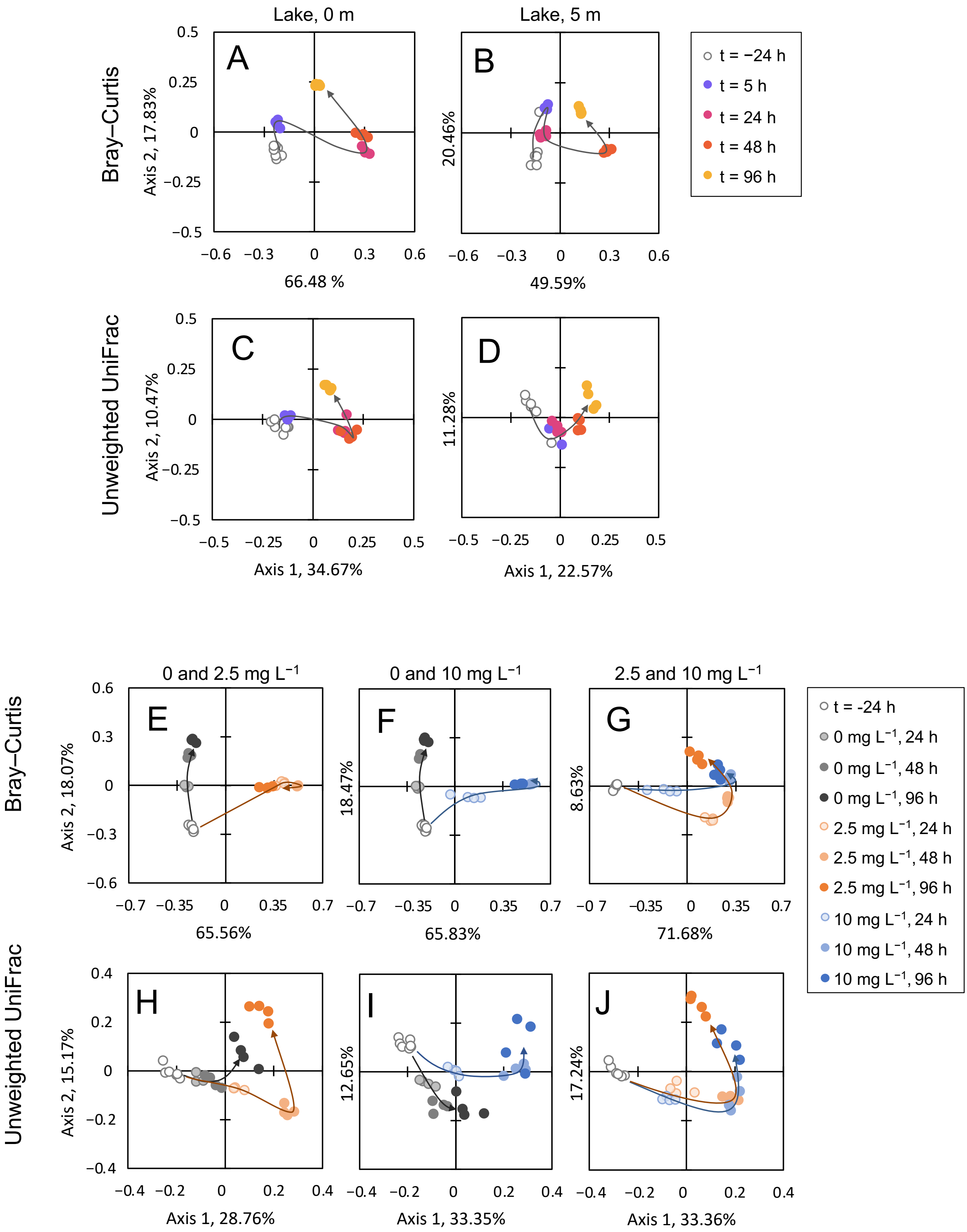

Figure S15. Beta diversity of the microbial community during the H_2O_2 treatment in June. PCoA plots of the beta diversity of the microbial community during (**A-D**) the lake treatment and (**E-J**) the incubation experiments in June. Beta diversity in (**A**,**B**) and (**E-G**) is based on the Bray-Curtis dissimilarity matrix, and in (**C**,**D**) and (**H-J**) on the unweighted UniFrac distance matrix. Each symbol in the graph represents one sample, all samples with symbols of the same color are biological replicates. Gray open circles represent samples in the lake prior to the H_2O_2 treatment (i.e., the starting community at t = -24 h). Arrows visualize the trajectories of the bacterial communities throughout the experiment. Pairwise PERMANOVAs revealed significant differences between all clusters within the same panel.

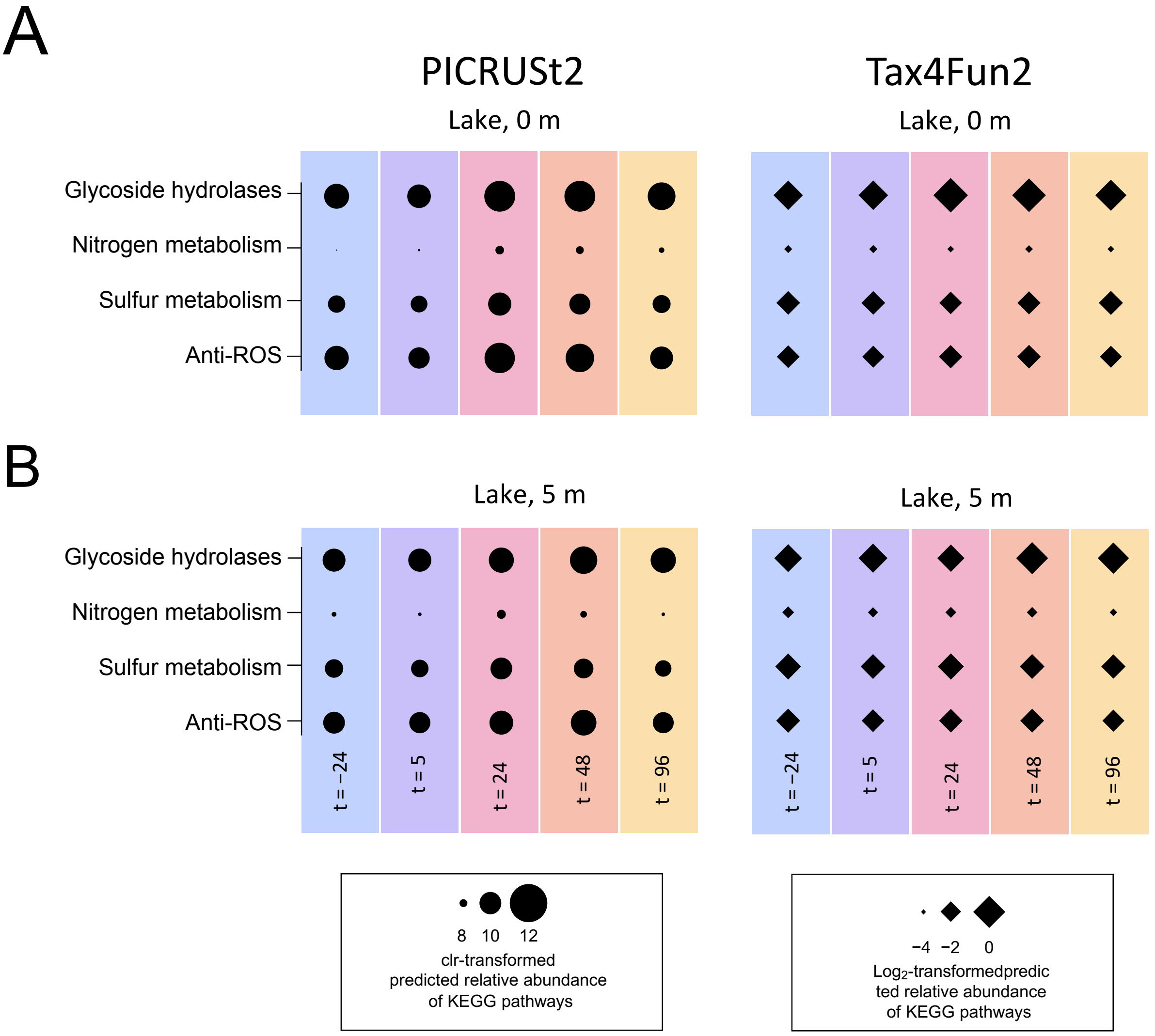

Figure S16. Functional prediction of the microbial community during the lake treatment in June. The graphs show predicted relative abundances of selected KEGG orthologs or pathways for glycoside hydrolases, nitrogen and sulfur metabolism and anti-ROS enzymes at (**A**) 0 m depth and (**B**) 5 m depth during the lake treatment in June. The relative abundances of selected KEGG orthologs or pathways are predicted by PICRUSt2 (circles) or Tax4Fun2 (triangles). The PICRUSt2 predictions are shown as clr-transformed counts while the Tax4Fun2 predictions are shown as log_2-transformed percentages. Columns show the average of the predicted relative abundances of *n*=7 biological replicates for the first time point (t = -24 h) at 0 m depth, *n*=6 biological replicates for the first time point at 5 m depth, and *n*=4 biological replicates for all other time points.

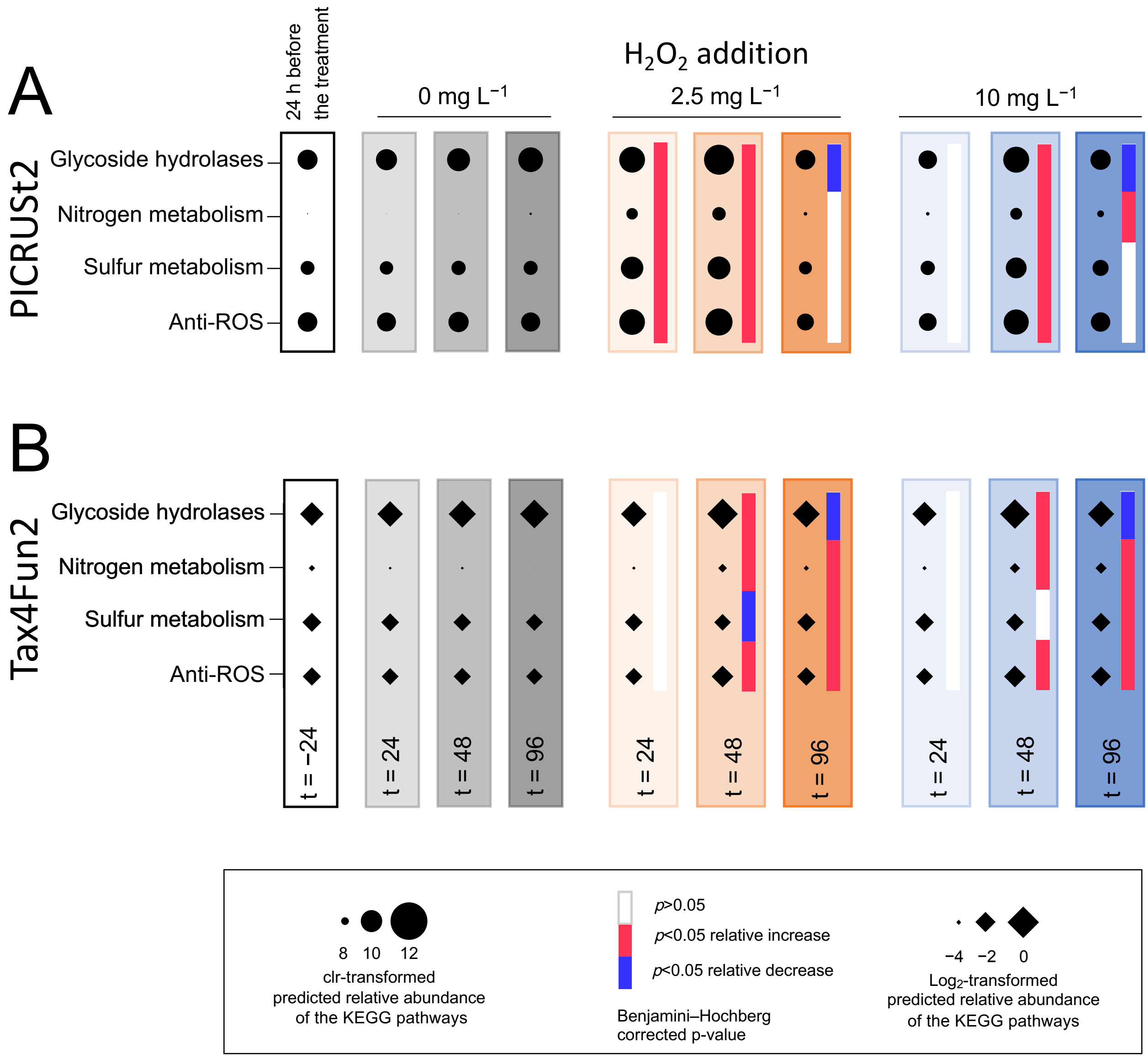

Figure S17. Functional prediction of the microbial community during the incubation experiments in June. The graphs show relative abundances of selected KEGG orthologs or pathways for glycoside hydrolases, nitrogen and sulfur metabolism and anti-ROS enzymes during the incubation experiments in June, as predicted by (**A**) PICRUSt2 and (**B**) Tax4Fun2. The first column presents relative abundances of the KEGG pathways in the lake prior to the incubation experiments (t = -24 h, with *n* = 7 biological replicates). The other columns present relative abundances in the incubation experiments at three subsequent time points (t = 24, 48 and 96 h, with *n* = 4 biological replicates per time point). Gray columns represent the control incubations (0 mg L^{-1} H_2O_2), orange columns the 2.5 mg L^{-1} H_2O_2 treated incubations, and blue columns the 10 mg L^{-1} H_2O_2 treated incubations. The color intensities of the columns represent different time points. Relative abundances are shown as (**A**) clr-transformed counts for PICRUSt2 and (**B**) log_2-transformed percentages for Tax4Fun2. Significant differences in relative abundances, at the same time point, between treated and control incubations were tested using (**A**) ALDEx2 and (**B**) a Wilcoxon test (see main text for details). Significant increases compared to the control are shown in red and significant decreases in blue, in the heatmap next to each time point. White fields in the heatmap indicate that relative abundances were not significantly different between the treated and control incubations.

Table S1. Weather data during the treatments in June and August. T_{max} = daily maximum air temperature.

Treatment	Timepoint	T_{max} (°C)	Max. wind gust (m/s)	Max. hourly mean wind speed (m/s)	Sunshine duration (h)	Daily precipitation amount (mm)	Precipitation duration (h)
June	t = -2 4	19.2	14	9	3.1	1.7	2.3
	t = 0	22.2	10	7	4.8	0	0
	t = 24	23.7	13	9	7.1	0	0
	t = 48	16.5	17	11	8.2	-1	0
	t = 72	16.1	17	11	1.4	-1	0
	t = 96	18.9	8	5	4	0	0
August	t = -2 4	30.4	8	4	13.9	0	0
	t = 0	33.7	15	7	10	2	1
	t = 24	23.5	15	10	5	3.5	1
	t = 48	22.8	23	10	1.8	7.5	4
	t = 72	22	18	10	7.6	16.4	6.2
	t = 96	20.4	12	9	10.9	0.4	0.2
